# Spatial and Annual Variation in Microbial Abundance, Community Composition, and Diversity Associated With Alpine Surface Snow

**DOI:** 10.3389/fmicb.2021.781904

**Published:** 2021-11-29

**Authors:** Lucas Fillinger, Kerstin Hürkamp, Christine Stumpp, Nina Weber, Dominik Forster, Bela Hausmann, Lotta Schultz, Christian Griebler

**Affiliations:** ^1^Department of Functional and Evolutionary Ecology, University of Vienna, Vienna, Austria; ^2^Institute of Radiation Medicine, Helmholtz Zentrum München, Neuherberg, Germany; ^3^Institute of Groundwater Ecology, Helmholtz Zentrum München, Neuherberg, Germany; ^4^Joint Microbiome Facility of the Medical University of Vienna and the University of Vienna, Vienna, Austria; ^5^Department of Laboratory Medicine, Medical University of Vienna, Vienna, Austria

**Keywords:** alpine cryobiosphere, microbial biogeography, virus-like particles, VLP, European Alps, Jungfraujoch, Sonnblick, Zugspitze

## Abstract

Understanding microbial community dynamics in the alpine cryosphere is an important step toward assessing climate change impacts on these fragile ecosystems and meltwater-fed environments downstream. In this study, we analyzed microbial community composition, variation in community alpha and beta diversity, and the number of prokaryotic cells and virus-like particles (VLP) in seasonal snowpack from two consecutive years at three high altitude mountain summits along a longitudinal transect across the European Alps. Numbers of prokaryotic cells and VLP both ranged around 10^4^ and 10^5^ per mL of snow meltwater on average, with variation generally within one order of magnitude between sites and years. VLP-to-prokaryotic cell ratios spanned two orders of magnitude, with median values close to 1, and little variation between sites and years in the majority of cases. Estimates of microbial community alpha diversity inferred from Hill numbers revealed low contributions of common and abundant microbial taxa to the total taxon richness, and thus low community evenness. Similar to prokaryotic cell and VLP numbers, differences in alpha diversity between years and sites were generally relatively modest. In contrast, community composition displayed strong variation between sites and especially between years. Analyses of taxonomic and phylogenetic community composition showed that differences between sites within years were mainly characterized by changes in abundances of microbial taxa from similar phylogenetic clades, whereas shifts between years were due to significant phylogenetic turnover. Our findings on the spatiotemporal dynamics and magnitude of variation of microbial abundances, community diversity, and composition in surface snow may help define baseline levels to assess future impacts of climate change on the alpine cryosphere.

## Introduction

The alpine cryosphere is among the ecosystems that are the most affected by climate change. In regions like the European Alps, surface air temperatures have increased at a faster rate over the past decades compared to the global average (Hock et al., 2019). The total glacier volume in the European Alps is predicted to decrease by 63–94% compared to present-day levels by the end of this century (Zekollari et al., 2019). Snow cover duration and snow mass are projected to decline by 30–80% and 10–40%, respectively, over the same time period, while winter precipitation extremes will occur more frequently. Overall, these changes are expected to alter the amount, quality, and seasonality of runoff (Hock et al., 2019). As mountain snow and ice are important sources of freshwater for downstream environments like rivers and lakes, these changes can have far reaching consequences for biogeochemical processes and biodiversity on a larger scale (Boetius et al., 2015; Hotaling et al., 2017; Hock et al., 2019; Ren et al., 2019; Zhou et al., 2019; Elser et al., 2020).

Microbial communities play a key role in the nutrient composition of mountain surface ice and snowpack. Despite extreme conditions characterized by low temperatures and intense UV radiation, microbial growth and metabolic activity have repeatedly been documented in such environments (Carpenter et al., 2000; Anesio et al., 2010; Stibal et al., 2012b; Lopatina et al., 2013), supporting primary production via fixation of atmospheric carbon and nitrogen, and heterotrophic processing of nutrients produced *in situ* and deposited from the atmosphere as dust (Stibal et al., 2012a; Maccario et al., 2015, 2019; Antony et al., 2016; Franzetti et al., 2016; Ren et al., 2019; Zhou et al., 2019). Additionally, viruses have been proposed to be an important factor in the regulation of nutrient cycles by modulating host metabolism and releasing labile organic matter from microbial cells through lysis (Anesio and Bellas, 2011; Zhong et al., 2021). Most of these observations stressing the importance of viruses in snow and ice come from cryoconite holes of arctic glaciers (Anesio et al., 2007; Anesio and Bellas, 2011; Maccario et al., 2015), where viral lysis has been estimated to release about one third of the carbon from microbial production (Rassner et al., 2016). Organic matter produced and processed in the alpine cryosphere is transported downstream with meltwater, together with microbial cells and viruses, which can be infectious also for cells in meltwater-fed aquatic environments (Anesio et al., 2007). Hence, the functioning and activity of microorganisms as well as microbial and viral abundances in the alpine cryosphere can have an impact on nutrient levels and stoichiometries in other environments downstream (Ren et al., 2019; Zhou et al., 2019).

Characterizing microbial and viral abundances as well as microbial community composition and diversity associated with surface snow and ice therefore is an important step toward a better understanding of current and future changes in the alpine cryosphere, and concomitant effects on meltwater-fed environments. Previous studies have identified atmospheric deposition as the main mechanism that seeds mountain snow surfaces with microbial cells (Boetius et al., 2015). The frequency and intensity of this deposition is spatiotemporally variable, for instance depending on the occurrence of dust events, which in turn can result in variation of microbial cell abundances, and cause significant differences in community composition across space and time (Segawa et al., 2005; Chuvochina et al., 2011; Meola et al., 2015; Els et al., 2019; Courville et al., 2020). Following deposition, microbial taxa are subject to selection imposed by environmental factors such as high solar radiation as well as low temperatures and nutrient levels, which only allow a certain fraction of the initially deposited taxa to persist and remain active (Segawa et al., 2005; Chuvochina et al., 2011; Lopatina et al., 2013; Els et al., 2019, 2020; Maccario et al., 2019).

**FIGURE 1:**
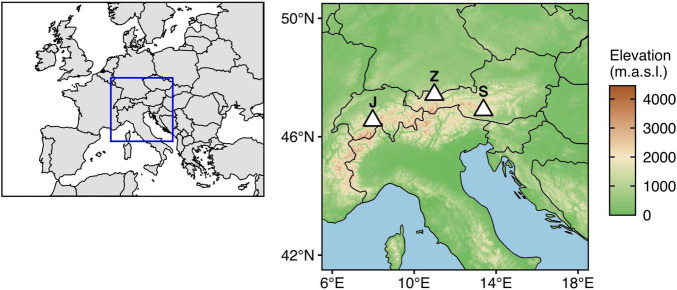
Geographic locations of the study sites (*J*: Jungfraujoch, 3572 m.a.s.l.; *Z*: Zugspitze, 2420 m.a.s.l.; *S*: Sonnblick, 3106 m.a.s.l.). Shapefiles for country borders were retrieved from Natural Earth (https://www.naturalearthdata.com/). Topographic data were retrieved from ETOPO1 Global Relief Model (Amante and Eakins, 2009) using metR (v. 0.9.2; Campitelli, 2021).

Knowledge on dynamics and the magnitude of variation in microbial abundance, community composition, and diversity is necessary for establishing a baseline of natural variation against which future changes can be compared in order to assess climate change impacts. Recording spatiotemporally data is an important step toward achieving this goal. However, so far, studies on microbial communities associated with mountain surface snow and ice have either focused on temporal changes at single locations or small spatial scales (Segawa et al., 2005; Chuvochina et al., 2011; Pittino et al., 2018; Els et al., 2019, 2020), or differences between locations at single time points (Wunderlin et al., 2016; Azzoni et al., 2018; Brown and Jumpponen, 2019), whereas data covering both spatial and temporal variation are scarce. As a first initiative to fill this gap, we collected samples across depth profiles of seasonal snowpack at the end of snow accumulation periods in two consecutive years (2015 and 2016) from three mountain summits along a longitudinal transect across the European Alps (i.e., Jungfraujoch, Switzerland; Zugspitze, Germany; Sonnblick, Austria) (Figure 1). In this study, we report spatiotemporal variation in abundances of prokaryotic cells and virus-like particles (VLP) measured by flow cytometry in addition to differences in microbial community composition and diversity inferred from 16S rRNA gene amplicon sequence variants (ASVs).

## Materials and Methods

### Site Descriptions and Sample Collection

The Jungfraujoch site was located in the Swiss Alps (46.5475°N, 7.9851°E, 3572 m.a.s.l.) directly south of the High Altitude Research Station Jungfraujoch in the firn of the Aletsch glacier. Mean air temperatures are −7.2°C with solid precipitation and strong winds throughout the year. Sampling campaigns were conducted on June 25, 2015, and June 1, 2016. Mean snow water equivalents (SWE) in 2015 and 2016 were 1994 mm and 1408.6 mm, respectively.

The Sonnblick is one of the highest mountains in the Austrian Alps (47.0536°N, 12.9578°E, 3106 m.a.s.l.) belonging to the Goldberg mountain range. Mean air temperatures are −5.1°C with an average precipitation of 2263 mm. The area is strongly wind exposed and snowfall occurs throughout the whole year. The sampling location was situated at the northern margin of the Kleinfleißkees glacier on a small rock outcrop, directly south of the mountain summit close to the Sonnblick Observatory research station. Sampling campaigns were conducted on July 2, 2015, and June 8, 2016. Mean SWE in 2015 and 2016 were 1587 mm and 2125 mm, respectively.

The Zugspitze situated in the southern Bavarian Alps is Germany’s highest mountain (47.4211°N, 10.9854°E, 2420 m.a.s.l.). Underneath the summit and surrounded by a horseshoe-shaped ridge of further summits, a flat karstic plateau serves as a popular skiing area and drains the whole Zugspitze region to the Partnach river in the east. The area is strongly wind exposed and receives intense solar radiation due to its south-eastern exposition. The average amount of precipitation is 2085 mm with liquid precipitation only during <10 weeks in summer. Mean air temperatures are −4.8°C. The sampling location was selected in the central western part of the plateau in a fenced area used by the Bavarian Avalanche Warning Service and situated close to the Environmental Research Station Schneefernerhaus (further details on the site are described in Hürkamp et al., 2019). Sampling campaigns were conducted on May 13, 2015, and May 3, 2016. Mean SWE in 2015 and 2016 were 1222 mm and 1521 mm, respectively.

The sampling locations at all three sites were selected to provide a flat rock surface to avoid potential interflow on sloping snow layer boundaries. The sampling location at each study site was the same in both years. To ensure that the sampled snow profiles covered the whole seasonal snowpack, vertical snow pits at Zugspitze and Sonnblick were dug all the way to the bottom on rock outcrops that had been free of snow cover during the previous summer season. At Jungfraujoch, the profiles were confined by a massive ice layer at the bottom, preventing further digging. Descriptions of the snow structures at the different layers according to Fierz et al. (2009) are listed for all sites in Supplementary Tables 1–6. Snow density and SWE were measured directly in the field by determination of snow mass of a defined volume using a snow cylinder.

Samples for the quantification of prokaryotic cells and VLP were collected separately into sterile 50 mL Falcon tubes at ∼10 cm intervals, taking into account differences in snow texture. Samples for cell and VLP counting were fixed on-site with 2.5% v/v and 0.5% v/v glutardialdehyde (final concentration), respectively. Vertical slot samples were taken over the total depth of a snow layer or divided into more than one sample for layers >20 cm. For layers with a thickness <5 cm, for example thin ice layers as well as the entire profile at Zugspitze in 2015, horizontal slots of about 10 cm in length were sampled at the center of each layer parallel to the snow surface. Samples for stable isotope analyses were collected into 50 mL polyethylene bottles at the same positions as samples for prokaryotic cell and VLP counting. The bottles were kept closed during snow melting to prevent isotope fractionation for example due to evaporation. Slot samples for DNA extraction were collected from selected layers of up to 100 cm in sterile 2 L wide neck polyethylene bottles. In general, the sample volume was reduced to ∼10% of the initially sampled snow volume after melting. Samples were kept cooled in polystyrene boxes for transport to the lab. Samples for prokaryotic cell counting and isotope analyses were kept at 4°C until analysis; samples for VLP counting and DNA extraction were stored at −20°C.

### Determination of Prokaryotic Cell and Virus-Like Particle Numbers by Flow Cytometry

Numbers of prokaryotic cells and VLP were measured separately in 500 μL snow meltwater aliquots. Samples were mixed with 500 μL suspension of reference beads as internal standard (Trucount Tubes; Becton-Dickinson, Franklin Lakes, NJ, United States). Fluorescent nucleic acid staining was used to distinguish cells and VLP from inorganic particles. Prokaryotic cells were stained with 1x (final concentration) SYBR Green I (Invitrogen, Darmstadt, Germany) during incubation at room temperature for 10 min in the dark. VLP were stained with 1x (final concentration) SYBR Gold (Invitrogen) during incubation at 80°C for 5 min followed by 10 min at room temperature in the dark. Cells and VLP were counted in duplicate on a FC500 CYTOMICS flow cytometer equipped with a 488 nm argon ion laser (Beckman Coulter, Brea, CA, United States).

### Determination of Stable Isotope Ratios

Comparing stable isotopes of oxygen (δ^18^O) and hydrogen (δ^2^H) in snow with the isotopic composition in precipitation provides information on potential transport processes within the accumulated snowpack and the temporal origin of the snow (Moser and Stichler, 1974). For example, lower isotope ratios in snow are associated with precipitation origination from colder temperatures or months. Additionally, freezing, thawing, water vapor exchange, and sublimation processes can influence the isotopic composition of the snow (Stichler, 1987; Gat, 1996).

δ^18^O and δ^2^H were analyzed in melted snow samples using laser spectroscopy (Picarro L2120-i and Picarro L2140-i, Picarro, Santa Clara, CA, United States). Stable isotope compositions in precipitation and snow are presented in delta notation as δ-value (‰). Precision of the instrument (1σ) was better than 0.6‰ and 0.15‰ for δ^2^H and δ^18^O, respectively. For comparisons of the isotopic composition of the snow profiles with precipitation, monthly or biweekly data were taken from nearby national monitoring sites at Zugspitze (own data), Jungfraujoch (personal communication: M. Leuenberger, High Altitude Research Station Jungfraujoch, February 9, 2016), and Feuerkogel as the closest station to Sonnblick (Environment Agency Austria [EAA], 2016; Supplementary Table 7). Elevation gradients were used to account for differences in the isotope ratios due to different altitudes of isotope monitoring sites and snow profiles. Winter precipitation data were normalized to minimize differences in the amounts of precipitation and snow, and SWE (Hürkamp et al., 2019). Snow accumulation periods for different depth zones were approximated from the comparison of the normalized data. Larger, unexpected differences between isotopic composition in precipitation and snow profiles can be caused by redistribution of the snowpack, for example caused by wind drift. Only δ^18^O values are reported here due to the linear relationship between δ^18^O and δ^2^H and because fractionation processes due to sublimation are of minor importance as shown for Zugspitze by Hürkamp et al. (2019).

### DNA Extraction

Snow meltwater samples were pooled from several adjacent layers to an equal volume of 2 L per sample. Cells for DNA extraction were collected from snow meltwater on 0.2 μm polycarbonate membrane filters (Merck-Millipore, Carrigtwohill, Ireland). DNA was extracted from the filters using bead beating and phenol–chloroform extraction as described by Pilloni et al. (2012). DNA was precipitated with 30% w/v polyethylene glycol containing 3 M NaCl for 2 h at 4°C followed by centrifugation at 20,000 × *g* for 30 min. The DNA pellet was washed twice with 150 μL 80% v/v ice-cold ethanol and resuspended in 15 μL EB buffer (Qiagen, Hilden, Germany). DNA was stored at −80°C.

### 16S rRNA Gene Amplicon Sequencing and Data Processing

The V4 region of the 16S rRNA gene was amplified using a unique dual barcoding two-step PCR approach (UDB-H12) as described by Pjevac et al. (2021) with primers 515F (Parada et al., 2016) and 806R (Apprill et al., 2015). Amplicons were sequenced in paired-end mode (2 × 300 bp) on a MiSeq platform (Illumina, San Diego, CA, United States) at the Joint Microbiome Facility of the Medical University of Vienna and the University of Vienna under project ID JMF-2006-1. Sequence data were processed in R (version 3.6.1; R Core Team, 2021) using DADA2 (v. 1.14.1; Callahan et al., 2016a) following the workflow by Callahan et al. (2016b). ASVs were inferred across all samples in pooled mode (further details on sequence trimming and settings for quality filtering are described in Pjevac et al., 2021). Taxonomic assignment was done by mapping ASV sequences against the SILVA SSU reference database (release 138; McLaren, 2020) using the RDP Naive Bayesian Classifier implemented in the DADA2 “assignTaxonomy” function with default confidence settings (i.e., minimum bootstrap confidence set to 50). ASVs classified as unclassified domain, eukaryotes, mitochondria, or chloroplasts were discarded from the dataset, as well as samples with less than 100 sequence reads. The final ASV table used for downstream analyses contained 427 ASVs with numbers of reads per sample ranging between a minimum and maximum of 214 and 9172, respectively (median: 1549; mean: 2773.2). For phylogenetic community analyses, sequences were aligned using the “AlignSeqs” function of the DECIPHER package (v. 2.16.1; Wright, 2016). A phylogenetic tree was constructed from the alignment using FastTree with a GTR + CAT model (v. 2.1.11; Price et al., 2010), and subsequently midpoint-rooted in R using the phytools package (v. 0.7.80; Revell, 2012). The 16S rRNA gene amplicon sequence data are publicly available at the NCBI Sequence Read Archive (accession number PRJNA756880).

### Data Analysis

All analyses were done in R (v. 4.0.2). Differences in numbers of prokaryotic cells, VLP, and VLP-to-prokaryotic cell ratios between sites within years were tested using Kruskal–Wallis tests and Dunn’s *post hoc* test for pairwise comparisons with Bonferroni *p*-value adjustment for multiple testing using the PMCMRplus package (v. 1.9.0; Pohlert, 2021). Differences between years within sites were tested using Wilcoxon rank sum tests.

Taxonomic and phylogenetic microbial community alpha diversity were estimated based on Hill numbers of orders *q* = 0 (taxon richness/Faith’s phylogenetic diversity), *q* = 1 (exponential of Shannon entropy/phylogenetic entropy), and *q* = 2 (Gini-Simpson index/Rao’s Q) inferred from interpolation and extrapolation of rarefaction curves (Chao et al., 2014a,b). To account for differences in the number of sequence reads between samples, diversity was calculated for all samples at an equal coverage level of 92.8%, which was the highest possible level for this dataset based on twice the smallest reference sample size (Chao et al., 2014b). Taxonomic and phylogenetic Hill numbers were calculated using the iNEXT (v. 2.0.20; Chao et al., 2014b) and iNextPD (v. 0.3.1; Hsieh and Chao, 2017) packages, respectively.

For calculating microbial community beta diversity, ASV abundances were converted to relative abundances within samples. Taxonomic and phylogenetic beta diversity were calculated as Bray–Curtis dissimilarity and relative abundance-weighted β-mean pairwise distance (β-MPD) using the vegan (v. 2.5-7; Oksanen et al., 2020) and picante (v. 1.8.2; Kembel et al., 2010) packages, respectively. PCoA ordinations for visualizing differences in community composition were generated using the ape package with Lingoes correction for negative eigenvalues (v. 5.4.1; Paradis and Schliep, 2019). Differences in community composition between sites and years were assessed using PERMANOVA (“adonis2” function, vegan) with 999 permutations for significance testing.

We took precautions to ensure that results of the beta diversity analyses obtained from relative abundance data were not biased by the variation in the number of sequence reads between samples. Bray–Curtis dissimilarity and β-MPD were additionally calculated on 100 rarefied ASV abundance tables that were generated in independent iterations of random subsampling without replacement (“rrarefy” function, vegan) to 214 reads per samples (i.e., the smallest number of reads per sample across all samples in the dataset). For both beta diversity measures, we found strong agreement between the values obtained from non-rarefied relative abundances and those obtained from rarefied abundance data based on Mantel correlation analyses and Gaussian generalized linear regression (Supplementary Figure 1). Furthermore, all PERMANOVA tests were additionally performed on each of the 100 Bray–Curtis dissimilarity and β-MPD matrices, respectively, calculated with the rarefied abundances. In all cases, results regarding effect sizes and significance of explanatory variables were highly similar and led to identical conclusions as those obtained from tests based on non-rarefied relative abundance data (Supplementary Figures 2–5). Finally, we directly tested for the effect of differences in the number sequence reads on the observed beta diversity patterns obtained from relative abundance data by repeating the PERMANOVA tests including the number of reads per sample as additional explanatory variable, following the recommendation by Weiss et al. (2017). The number of sequence reads did not have a significant effect in any of the analyses after controlling for site or year (all partial *R*^2^ < 0.08; all *p* > 0.18; results not shown), and were therefore not considered further for the final analyses presented below.

## Results

### Snow Profile Characteristics and Dynamics of Prokaryotic Cell and Virus-Like Particle Numbers

**FIGURE 2:**
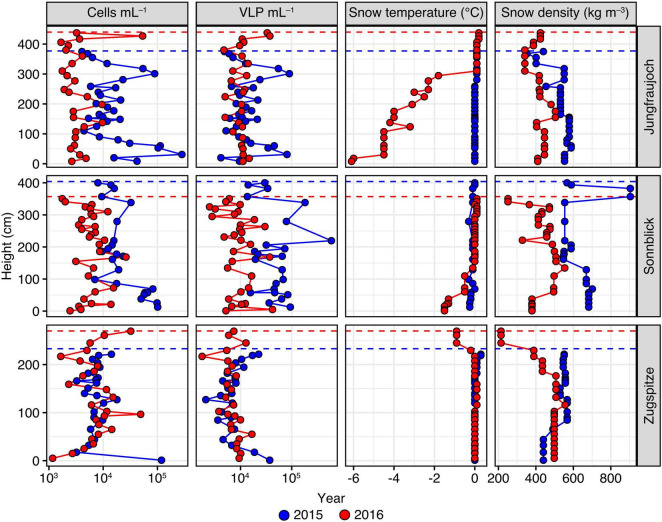
Numbers of prokaryotic cells and VLP per mL snow meltwater, in addition to snow temperature and snow density measured across the snowpack profiles per site and year. Height of the snow layer is given in cm above the bottom of the snowpack. Dashed lines represent the maximum snowpack height for each year.

The snowpack at Jungfraujoch in 2015 was 377 cm deep and was confined by a massive ice layer at the bottom. Near isothermal conditions with temperatures around 0°C across the entire profile indicated the end of the snowpack ripening and beginning of the melting phase. Several thin ice layers occurred throughout the depth profile, resulting in snow densities of 353–588 kg m^–3^ (Figure 2). A sandy layer was observed at ∼260 cm above ground (Supplementary Table 3). In 2016, snowpack height was 440 cm, with snow densities ranging between 341 and 544 kg m^–3^. Sandy layers were observed at heights of ∼20 cm, ∼130 cm, and ∼190 cm above ground (Supplementary Table 6).

At Sonnblick, snowpack height in 2015 was 404 cm with several thin ice layers in between. Snow densities were relatively high, especially in the top layers, ranging between 507 and 682 kg m^–3^. A mineral-rich, brown layer was visible at a height of ∼70 cm, which likely was an old surface layer possibly derived from the previous summer season (Supplementary Table 2). In 2016, the snowpack height was 357 cm and the snow was less compacted. The top 30 cm consisted of fresh snow with a lower density compared to deeper layers. The overall snow density ranged between 251 and 554 kg m^–3^. A visible sand layer was observed at ∼220 cm above ground, which was proven to originate from a Sahara dust event that occurred on April 4, 2016 (Supplementary Table 5).

At Zugspitze, snowpack height in 2015 was 233 cm with snow densities ranging between 440 and 567 kg m^–3^. In 2016, snowpack height was 270 cm. The profile consisted of a less dense, fresh snow layer of ∼50 cm at the top; overall snow densities across the whole profile ranged between 214 and 589 kg m^–3^. A sandy layer was observed at ∼180 cm above ground that derived from a Sahara dust event occurring on April 6, 2016 (Supplementary Tables 1, 4).

Numbers of prokaryotic cells and VLP showed significant variation between sites and years, albeit over a relatively modest range of one order of magnitude in general (Figure 2). Across all samples, the median number of prokaryotic cells was 7.41 × 10^3^ per mL of snow meltwater with an interquartile range (IQR) of 9.98 × 10^3^. For both years, we found significant differences in cell numbers between sites (Kruskal–Wallis rank sum tests; 2015: *H* = 20.2, *p* < 0.0001; 2016: *H* = 16.5, *p* = 0.0003). Closer inspection of these differences by pairwise comparisons using Dunn’s *post hoc* tests showed that in 2015 prokaryotic cell numbers at Zugspitze (median = 7.35 × 10^3^ cells mL^–1^, IQR = 2.82 × 10^3^) were significantly lower compared to Jungfraujoch (median = 1.19 × 10^4^ cells mL^–1^, IQR = 1.48 × 10^4^, *p* = 0.011) and Sonnblick (median = 1.77 × 10^4^ cells mL^–1^, IQR = 3.91 × 10^3^, *p* < 0.0001). In 2016, cell numbers were significantly lower at Jungfraujoch (median = 2.98 × 10^3^ cells mL^–1^, IQR = 1.64 × 10^3^) compared to Sonnblick (median = 5.96 × 10^3^ cells mL^–1^, IQR = 3.13 × 10^3^, *p* = 0.0037) and Zugspitze (median = 6.81 × 10^3^ cells mL^–1^, IQR = 6.05 × 10^3^, *p* = 0.0005). Within sites, cell numbers were significantly higher in 2015 compared to 2016 at Jungfraujoch (Wilcoxon rank sum test; *W* = 724, *p* << 0.0001) and Sonnblick (*W* = 646.5, *p* << 0.0001).

Numbers of VLP were in a similar range as prokaryotic cell numbers (overall median = 9.90 × 10^3^ VLP mL^–1^, IQR = 1.00 × 10^4^) and showed significant variation between sites within years (Kruskal-Wallis rank sum tests; 2015: *H* = 36.2, *p* < 0.0001; 2016: *H* = 10.5, *p* = 0.0053). Multiple pairwise comparisons showed that these differences were due to significantly higher VLP numbers at Sonnblick in 2015 (median = 4.66 × 10^4^ VLP mL^–1^, IQR = 4.49 × 10^4^) compared to Jungfraujoch (median = 1.17 × 10^4^ VLP mL^–1^, IQR = 1.22 × 10^4^, *p* = 0.0002) and Zugspitze (median = 6.96 × 10^3^ VLP mL^–1^, IQR = 4.28 × 10^3^, *p* << 0.0001), while in 2016 VLP numbers were significantly lower at Zugspitze (median = 6.85 × 10^3^ VLP mL^–1^, IQR = 2.79 × 10^2^) compared to Jungfraujoch (median = 1.09 × 10^4^ VLP mL^–1^, IQR = 2.14 × 10^3^, *p* = 0.0036) but not Sonnblick (median = 9.31 × 10^3^ VLP mL^–1^, IQR = 6.03 × 10^3^, *p* = 0.24). Significant differences within sites between years were only found for Sonnblick (Wilcoxon rank sum test; *W* = 652, *p* << 0.0001).

Although VLP and prokaryotic cell numbers also varied across the depth of the snow profiles, there was no significant monotonic relationship between either parameter and height of the snow layer above bottom across all samples (cell numbers: Spearman’s *rho* = −0.140, *p* = 0.0810; VLP: *rho* = −0.150, *p* = 0.061). When tested within sites and years, significantly decreasing cell numbers with snow layer height were found only at Jungfraujoch (*rho* = −0.402, *p* = 0.0274) and Sonnblick (*rho* = −0.712, *p* = 0.0001) in 2015 (all others: | *rho*| < 0.14, *p* > 0.37). VLP numbers showed a significant decrease with snow layer height only at Zugspitze in 2016 (*rho* = −0.482, *p* = 0.0147; all others: | *rho*| < 0.31, *p* > 0.096).

Numbers of prokaryotic cells and VLP were moderately positively correlated only across all samples without distinguishing between sites and years (*rho* = 0.472, *p* << 0.0001). However, this relationship was not generally observed when considering the data within sites and years separately, where significant positive correlations were observed only at Jungfraujoch in 2015 (*rho* = 0.880, *p* << 0.0001) and Sonnblick in 2016 (*rho* = 0.389, *p* = 0.034) (Supplementary Figure 6).

**FIGURE 3:**
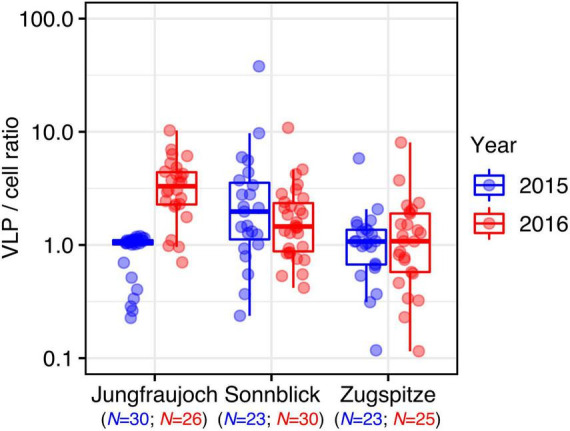
VLP-to-prokaryotic cell ratios per site and year.

Virus-like particle-to-prokaryotic cell ratios varied over two orders of magnitude (minimum: 0.12, maximum: 37.90) with an overall median of 1.24 (IQR = 1.37) (Figure 3). Differences within sites between years were observed only at Jungfraujoch (*W* = 77, *p* << 0.0001; all others: *W* > 270, *p* > 0.35). Within years, VLP-to-cell ratios were significantly higher at Sonnblick in 2015 and Jungfraujoch in 2016 compared to the other two respective sites (*p* < 0.003). We further analyzed correlations between VLP-to-cell ratios and numbers of prokaryotic cells and VLP, respectively, to test whether changes in VLP-to-cell ratios were caused by changes in either only cell numbers or VLP alone, or concomitant changes of both. In the majority of cases, VLP-to-cell ratios were significantly positively correlated with VLP numbers, and significantly negatively correlated with prokaryotic cell numbers. The only exception was observed at Jungfraujoch in 2015, where both numbers of prokaryotic cells and VLP were significantly negatively correlated with VLP-to-cell ratios (Supplementary Figure 7).

In summary, we found significant albeit relatively small variation (within one order of magnitude) in the numbers of prokaryotic cells, VLP, and VLP-to-cell ratios between sites and years. However, we could not detect consistent patterns indicating generally increased numbers within a particular year or at any of the given sites. Moreover, occasional peaks in prokaryotic cell and VLP numbers were not always consistent with observed sandy layers in the snow profiles.

### Stable Isotope Ratios in Snow Profiles

**FIGURE 4:**
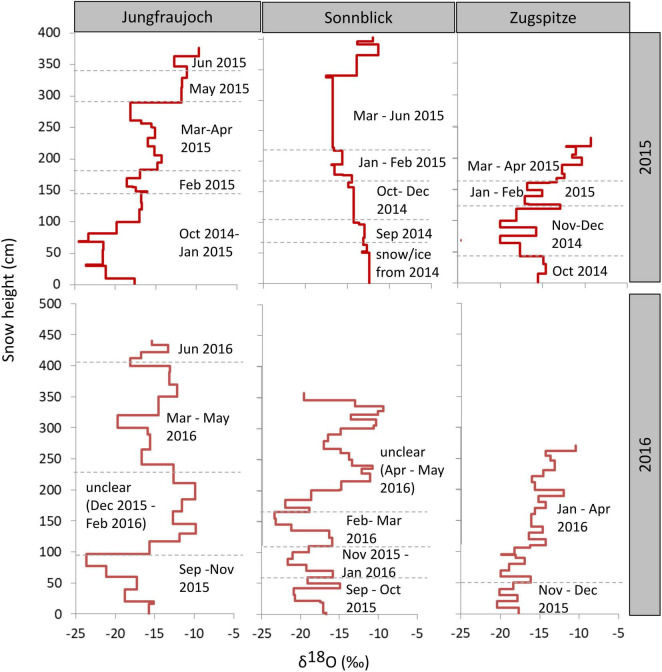
δ^18^O values across the snowpack profiles per site and year. Times of accumulation periods were approximated by considering isotope ratios in precipitation during the accumulation period as well as precipitation amount and snow water equivalents. Height of the snow layer is given in cm above the bottom of the snowpack.

To estimate approximate time points of snow accumulation (Figure 4), stable isotopes in snow were compared to isotope ratios measured in precipitation during snow accumulation periods (Supplementary Table 7). This was only possible by also considering the amount of snow in the snowpack calculated from individual depths and snow density (Figure 2), and the amount of precipitation (Supplementary Table 7). Overall, the annual snowpack at the three sites covered snow accumulation periods of about 6–10 months. Time differences between the oldest bottom layers and the youngest top layers from the previous season were approximately 4–8 months. It is worth mentioning that those are rough estimates, due to the coarse monthly or biweekly sampling of isotopes in precipitation and differences in snow and precipitation amounts. The dating of two snow layers in the snow profiles at Sonnblick and Zugspitze in 2016 could be verified by the presence of Saharan dust. The dust events occurred from April 4 to 6, 2016, (Supplementary Tables 4, S5) as proven by aerosol measurements of the German Federal Environment Agency (personal communication by L. Ries). Nevertheless, most of the isotope patterns from precipitation resembled the distribution in the snow profiles except for the middle and upper parts of profiles in 2016 from Jungfraujoch and Sonnblick, respectively. The isotope ratios between 0 and 100 cm and between 100 and 300 cm in the snowpack from Jungfraujoch in 2016 were about 2.5‰ lower and 5‰ greater, respectively, than expected from isotope ratios in precipitation, indicating either snow redistribution or melting (100–300 cm), and refreezing processes (0–100 cm). At Sonnblick in 2016, there were major differences in the water balance (450 mm more water in the snow profile than in precipitation). When comparing isotope ratios in the snow and precipitation, the difference in the water balance is associated with time points after March 2016 and snow accumulated >165 cm, still having almost similar isotope averages (−14.4‰ in precipitation and −14.1‰ in snow). This discrepancy can be explained by either erroneous precipitation amount analysis, or redistribution of snow due to wind drift.

### Dynamics of Microbial Community Composition and Diversity

**FIGURE 5:**
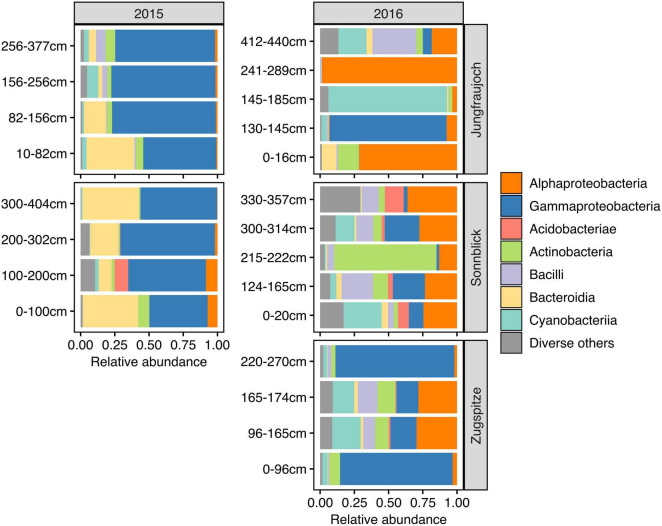
Microbial community composition across snowpack profiles per site and year based on relative abundances summarized at class level. The range of pooled layers used for DNA extraction is indicated in cm above the bottom of the snowpack. For clarity of display, classes with a combined relative abundance <5% across all samples are shown collectively as “Diverse others.”

**FIGURE 6:**
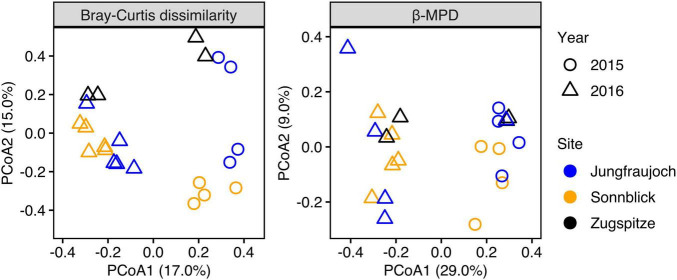
PCoA ordination plots of differences in taxonomic community composition based on Bray–Curtis dissimilarity calculated from relative ASV abundances, and differences in phylogenetic community composition calculated as relative abundance-weighted β-MPD.

Microbial communities displayed similar compositions within years across sites (Figure 5). In 2015, communities at Jungfraujoch and Sonnblick were dominated by Gammaproteobacteria (mainly *Massilia*, *Polaromonas*) and Bacteroidia (*Ferruginibacter*, *Hymenobacter*). These classes were replaced to a large extent in 2016 by Alphaproteobacteria (*Sphingomonas*, *Methylobacterium-Methylorubrum*, *Acidiphilum*), diverse Actinobacteria, Bacilli (*Staphylococcus*, *Streptococcus*), and diverse Cyanobacteria (abundances of the most dominant genera within the most abundant classes are shown in Supplementary Figures 8–13). In line with this qualitative observation, PCoA ordinations showed stronger clustering of samples by year than by site based on Bray–Curtis dissimilarity and especially β-MPD (Figure 6).

We assessed marginal effect sizes and significance of differences between sites and years using PERMANOVA considering only samples from Jungfraujoch and Sonnblick, since samples from both years were not available for the Zugspitze. The analysis showed that differences in taxonomic community composition measured as Bray–Curtis dissimilarity were significant between sites and years, although variation between years was larger [partial *R*^2^ = 0.1781, *F*_(1_,_17)_ = 3.67, *p* = 0.001] than between sites [partial *R*^2^ = 0.094, *F*_(1_,_17)_ = 1.94, *p* = 0.011]. However, differences in phylogenetic community composition based on β-MPD were significant only between years [partial *R*^2^ = 0.2255, *F*_(1_,_17)_ = 4.70, *p* = 0.001] but not between sites [partial *R*^2^ = 0.0543, *F*_(1_,_17)_ = 1.13, *p* = 0.26]. Comparable results were obtained from analyzing differences between all three sites considering only samples from 2016 [Bray–Curtis dissimilarity: *R*^2^ = 0.2522, *F*_(2_,_13)_ = 1.86, *p* = 0.001; β-MPD: *R*^2^ = 0.1822, *F*_(2_,_13)_ = 1.23, *p* = 0.131]. Taken together, the analyses indicate that microbial communities were composed of phylogenetically similar ASVs across sites within years, and that differences between sites were due to shifts in abundances of ASVs within phylogenetically similar clades.

**FIGURE 7:**
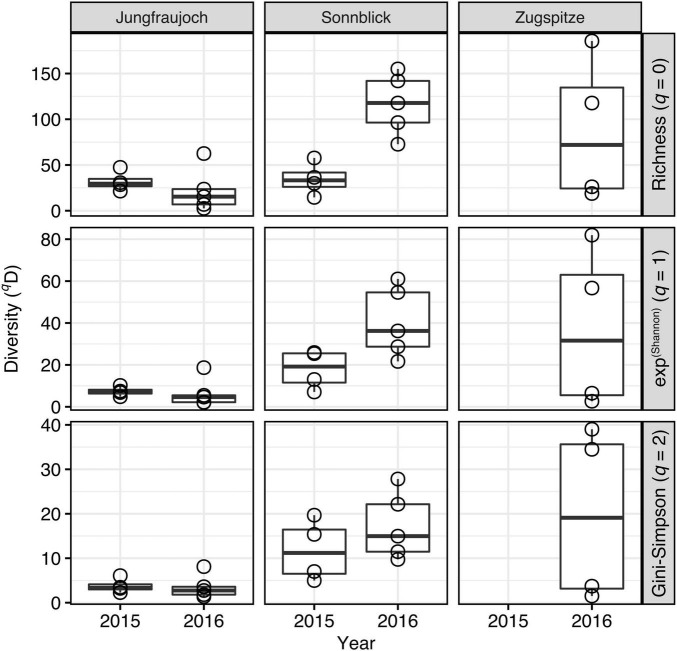
Microbial community alpha diversity estimates inferred from Hill numbers per site and year.

Microbial community alpha diversity inferred from Hill numbers generally decreased with higher orders of *q*, indicating relatively low contributions of common (*q* = 1) and abundant ASVs (*q* = 2) to the total ASVs richness (*q* = 0) in the communities (Figure 7). Across all samples, average diversity was 59.5, 22.0, and 11.1 (minima: 2.6, 2.1, 1.4; maxima: 186.0, 81.9, 39.0) for orders *q* = 0, *q* = 1, and *q* = 2, respectively. Significant differences between sites were only observed in 2016 with 5–8 times higher diversity at Sonnblick compared to Jungfraujoch for all orders of *q* (Wilcoxon rank sum tests; all *W* = 0, *p* < 0.008), while in 2015 no significant differences between sites were observed for any order of *q* (all *W* ≥ 1, *p* > 0.05) (samples from the Zugspitze were not included in these analyses due to the lack of samples from 2015, and the generally large variation and small number of samples for 2016). Although community alpha diversity showed some variation between years within sites, significant year-to-year variation was only observed at Sonnblick for *q* = 0 (*W* = 0, *p* = 0.016; all others: *W* ≥ 2, *p* > 0.28) but not for higher orders of *q*. Similar results were obtained based on phylogenetic community alpha diversity (Supplementary Figure 14). Thus, significant variation in community alpha diversity between sites appeared to have affected rare as well as common and abundant ASVs, whereas variation within sites between years was less pronounced, especially considering the more abundant fraction of the microbial communities.

Unfortunately, the relatively coarse pooling of samples that was necessary to ensure sufficient sample volumes and DNA yield did not allow for direct analyses of differences in microbial community composition or diversity in relation to snow layer height, time point of snow deposition inferred from stable isotopes, or snow temperature and density.

## Discussion

In this study, we set out to characterize spatiotemporal variation in microbial abundance, community composition, and diversity associated with seasonal surface snow in high altitudes of the European Alps, as a first step toward establishing a baseline for natural variation of microbial communities in these environments. Prokaryotic cells and VLP occurred in similar numbers ranging between 10^4^ and 10^5^ per mL of snow meltwater on average, with relatively little—albeit occasionally significant—variation between sites and years within one order of magnitude. These numbers are in a similar range as previously reported for alpine surface snow and ice (Segawa et al., 2005; Boetius et al., 2015; Lazzaro et al., 2015), including an earlier study at Jungfraujoch conducted in 2015 (Wunderlin et al., 2016), as well as numbers commonly found in cold, oligotrophic freshwater environments (Wommack and Colwell, 2000). In comparison to glacial cryoconite, these numbers are up to four orders of magnitude lower (Anesio et al., 2007; Bellas et al., 2013; Maccario et al., 2015), which is not surprising given the higher nutrient levels and microbial productivity in cryoconite compared to clean surface snow as investigated in our study.

Meola et al. (2015) have reported distinctly elevated abundances of prokaryotic cells in Sahara dust layers contained in surface snow collected at Jungfraujoch in 2014. While snow profiles in our study also contained visible sand layers likely derived from Sahara dust events, peaks with elevated numbers of prokaryotic cells or VLP compared to clean snow above or below were not always consistent with these layers. Similarly, we did not find a consistent trend regarding changes in prokaryotic cell and VLP numbers with snow layer height. The lack of a noticeable, consistent relationship between cell and VLP numbers, respectively, and observed dust layers or snow layer height in our study might be explained by findings by Lazzaro et al. (2015). Here, the authors showed that microbial cells are mobilized more readily than dust particles during intermediate snow melting, which can lead to homogenization of cells across the depth profile of snowpack along meltwater pores over time. Single instances where significantly increasing cell numbers toward the bottom of the snowpack were observed in our study (Jungfraujoch and Sonnblick, 2015) suggest accumulation of cells at greater depths due to snow melting and downward transport.

Since prokaryotic cells and VLP were present in similar numbers, VLP-to-cell ratios ranged slightly above 1 on average and rarely exceeded 10, even though the total variation spanned two orders of magnitude. Except for the considerable difference at Jungfraujoch between the 2 years, the variation between sites and years was comparatively low. Overall, variation of environmental VLP-to-prokaryotic cell ratios over several orders of magnitude are not uncommon (Parikka et al., 2017). However, compared to other oligotrophic environments or glacial cryoconite (Anesio et al., 2007; Rassner et al., 2016; Parikka et al., 2017), VLP-to-cell ratios observed in our snow samples were about one order of magnitude lower. VLP-to-cell ratios are generally expected to decrease with decreasing microbial productivity common for oligotrophic environments, and high viral decay for example caused by intense solar radiation (Wommack and Colwell, 2000; Parikka et al., 2017). Both factors likely play a larger role in clean surface snow compared to cryoconite, which might explain the lower ratios observed in our study. Comparatively high VLP-to-cell ratios and strong positive correlations between the numbers of prokaryotic cells and VLP in cryoconite holes and glacial ice have been suggested to indicate strong control of viruses over microbial communities in these environments (Anesio et al., 2007; Anesio and Bellas, 2011; Bellas et al., 2013). The relatively low VLP-to-cell ratios and generally weak or lack of correlations between cell and VLP numbers observed in our study seem to suggest that such a strong viral control might not apply to alpine surface snow. Nevertheless, the single instance of a strong positive correlation between cell and VLP numbers observed for Jungfraujoch in 2015, and the large variation of VLP-to-cell ratios within the individual snow profiles might indicate that the strength of viral control is dynamic even in these environments, and might change over relatively small time scales, for instance with changes in nutrient inputs from the atmosphere (Rassner et al., 2016).

Microbial communities were composed of similar groups of organisms as have been found in previous studies on mountain surface snow and ice in different parts of the globe (Segawa et al., 2005; Boetius et al., 2015; Maccario et al., 2015; Antony et al., 2016; Carey et al., 2016; Wunderlin et al., 2016; Azzoni et al., 2018; Zhou et al., 2019), including the European Alps (Chuvochina et al., 2011; Meola et al., 2015; Wunderlin et al., 2016; Azzoni et al., 2018; Courville et al., 2020; Els et al., 2020), and >10000-year-old Himalayan glacial ice (Zhong et al., 2021). Dominant genera included known psychrophilic or psychrotolerant, UV-resistant organisms typical for cryosphere environments such as heterotrophic *Polaromonas* (Gammaproteobacteria; Darcy et al., 2011) and *Hymenobacter* (Bacteroidia; Dai et al., 2009; Klassen and Foght, 2011; Sedláček et al., 2019), or photoautotrophs within the *Chroococcidiopsidaceae* (Cyanobacteria; Baqué et al., 2013). Additionally, communities contained more generalist organisms such as *Massilia* (Gammaproteobacteria), *Sphingomonas* (Alphaproteobacteria), *Streptococcus* and *Staphylococcus* (Bacilli), or *Ferruginibacter* (Bacteroidia), which are frequently encountered in temperate freshwater and soil, but have also been found in mountain snow and ice by others (Segawa et al., 2005; Meola et al., 2015; Zhou et al., 2019; Zhong et al., 2021). The composition of surface snow-associated microbial communities in alpine environments can differ from those found at lower altitudes in the Arctic or Antarctic (Boetius et al., 2015), in part likely due to contributions of additional sources of microorganisms absent from alpine regions such as seawater and brine (Harding et al., 2011; Maccario et al., 2019). However, certain organisms like *Polaromonas*, but also other Proteobacteria, Cyanobacteria, Actinobacteria, Firmicutes, and Bacteroidetes have frequently been observed in snowpack in alpine regions like in our study and the ones cited above, as well as in the Arctic and Antarctic (e.g., see Harding et al., 2011; Hell et al., 2013; Antony et al., 2016). This suggests a wide distribution of these taxa across broad geographical ranges and altitudes.

Similar to previous studies, we observed significant variation in microbial community composition between years (Chuvochina et al., 2011; Pittino et al., 2018; Els et al., 2020) and sites (Azzoni et al., 2018; Courville et al., 2020). However, our analyses showed that differences in community composition between years were stronger compared to differences between sites within years, especially considering phylogenetic community composition. Hence, despite differences in the presence and abundance of individual ASVs, the microbial communities at different sites were composed of phylogenetically similar taxa. This observation is in line with previous studies that found similar phylogenetic groups in mountain surface snow across different locations despite overall significant differences in community composition (Wunderlin et al., 2016; Brown and Jumpponen, 2019). Els et al. (2019, 2020) showed that microbial community composition in tropospheric air and precipitation can vary significantly over time, which in turn contributes to temporal variation in community composition in mountain surface snow as microbes are deposited from the atmosphere (see also Chuvochina et al., 2011).

Interestingly, the stable isotope analyses suggested that the time spans of snow accumulation periods covered by the snow profiles in each year (6–10 months) were usually larger than time differences between the youngest top layers of the old snowpack and the oldest bottom layers of new snow accumulation periods in the following year (4–8 months). We may speculate that the seasonal snow surfaces at the different sites in our study had been initially colonized by microorganisms from similar sources, such as atmospheric dust and aerosols of similar origins, and that the composition of these seed communities varied strongly between years. The subsequent community succession following the initial colonization could have been determined to some degree by the composition of the seed communities through priority effects (Fukami, 2015), as well as similar environmental conditions across sites selecting for phylogenetically similar organisms (Maccario et al., 2019; Els et al., 2020). In addition to selection, we can expect that communities were additionally subject to ecological drift, causing random extinction and arrival of individual ASVs, thus leading to the significant albeit comparatively small differences in taxonomic community composition between sites within years.

Our estimates of community alpha diversity were in agreement with those observed in previous studies at Jungfraujoch from 2014 (Meola et al., 2015) and 2015 (Wunderlin et al., 2016), and estimates reported for Sonnblick for three consecutive seasons between 2016 and 2017 (Els et al., 2020). In line with our results, communities in these studies contained approximately 50–250 distinct microbial taxa on average, with a similarly low contribution of abundant taxa to the total richness, resulting in low community evenness. Comparable ranges have further been reported for surface snow from different alpine regions spanning from Anatolia to the Himalayas (Azzoni et al., 2018), or the Australian Alps (Wunderlin et al., 2016) (however, about 5–10 times higher estimates have been reported for mountain surface snow in the Sierra Nevada, United States; Carey et al., 2016). The modest variation in community alpha diversity between years in our study, and in comparison to estimates from earlier studies at the same sites, indicates that community alpha diversity—in contrast to community composition—varies within a relatively narrow range from year to year. Taking into account a certain bias due to methodological differences in amplicon sequencing and sequence data processing between studies, collectively these results suggest that alpine surface snow environments may have similar microbial community diversity equilibria (also referred to as carrying capacity for species richness; Storch and Okie, 2019) across wide geographical ranges that are relatively stable over consecutive seasons.

The predicted decrease in snow cover duration and snow mass combined with higher frequencies of precipitation extremes in the alpine cryosphere due to climate change (Hock et al., 2019) can be expected to affect the spatiotemporal dynamics and magnitude of variation in the microbiological parameters observed over the relatively short timescale of our study. We may assume that shorter snow cover duration might leave less time for selection processes to operate on microbial communities in the snowpack, which in turn could amplify the impact of stochastic drift and thereby cause larger variation in microbial community composition and diversity between seasons as well as locations. Similarly, abundances of and dynamics between microorganisms and viruses may be affected by changes in the quantity and quality of precipitation. As variation increases, uncertainties in predictions of the amounts and types of microorganisms and viruses that are transported with meltwater to downstream environments would also increase. Hence, we argue that knowledge on the spatiotemporal dynamics of microbial communities associated with mountain snow and changes in the magnitude of variation over time is an important factor for our understanding of climate change impacts on the alpine cryosphere and beyond. Our study provides a first glimpse at the temporal variation in microbial abundance, community composition, and diversity between two consecutive seasons at the three investigated sites. However, longer time series over several seasons are certainly necessary to assess whether this variation remains stable around a certain baseline like the values reported here, or rather shows an increasing trend over longer time scales in the future.

## Conclusion

Our study suggests that numbers of prokaryotic cells and VLP as well as microbial community alpha diversity in alpine surface snow show relatively little variation across space and snow accumulation periods on average. This is further supported by the agreement between values observed in our study and those previously reported for the same sites from different time points, as well as observations from studies at different high altitude locations. Relatively low VLP-to-prokaryotic cell ratios, and mostly weak or lacking correlations between numbers of prokaryotic cells and VLP observed in our study may indicate a weaker viral control over microbial communities in clean alpine surface snow compared to glacial ice or cryoconite reported by others. However, more direct research on this issue would be required. While microbial community composition was comparable between sites within years, we showed that in contrast to prokaryotic cell numbers and microbial community alpha diversity, community composition was highly dynamic and changed significantly between years. Based on previous findings, it seems likely that the strong year-to-year variation in community composition could have been determined by differences in the composition of seed communities derived from the atmosphere and precipitation, although our data do not allow any direct inferences in that regard. All in all, our findings add to the knowledge on spatiotemporal dynamics and magnitude of variation of microbial abundances, community composition, and diversity in alpine surface snow. This knowledge may aid defining baselines to assess future impacts of climate change on the alpine cryosphere.

## Data Availability Statement

The datasets presented in this study can be found in online repositories. The names of the repository/repositories and accession number(s) can be found in the article/Supplementary Material.

## Author Contributions

LF analyzed the data and wrote the manuscript. KH organized and conducted the sampling campaigns and sample collection. CS analyzed the stable isotope data. NW performed the flow cytometry measurements and participated in sampling campaigns. DF did the DNA extraction. BH performed the bioinformatic processing of raw amplicon sequence data. LS contributed to the data curation and literature research. CG organized and supervised sample processing for microbial community analyses. All authors have contributed to and provided feedback on the manuscript.

## Conflict of Interest

The authors declare that the research was conducted in the absence of any commercial or financial relationships that could be construed as a potential conflict of interest.

## Publisher’s Note

All claims expressed in this article are solely those of the authors and do not necessarily represent those of their affiliated organizations, or those of the publisher, the editors and the reviewers. Any product that may be evaluated in this article, or claim that may be made by its manufacturer, is not guaranteed or endorsed by the publisher.
