# Supplementary material for: Spatial and Annual Variation in Microbial Abundance, Community Composition, and Diversity Associated With Alpine Surface Snow
Source: Front Microbiol. 2021 Nov 29;12:781904. doi: 10.3389/fmicb.2021.781904 (PMC8667604; doi:10.3389/fmicb.2021.781904)
Supplement: Supplementary file 1 [file Data_Sheet_1.PDF]

Supplementary Material

Supplementary Figures

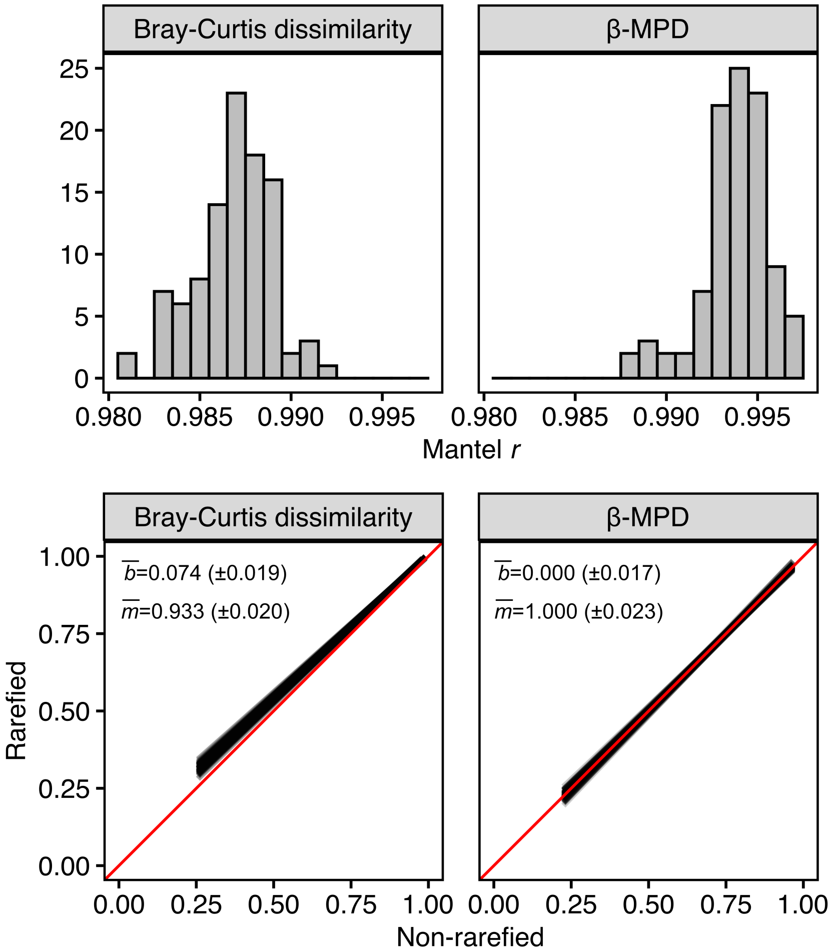

Figure S1: Comparison between microbial community beta diversity calculated with non-rarefied relative ASV abundances and rarefied abundances from 100 independent iterations of random subsampling without replacement to an equal number of 214 reads per sample. The top panel shows distributions of Mantel correlations between beta diversity calculated with non-rarefied relative abundances and rarefied abundances. The bottom panel shows linear regression curves from Gaussian generalized linear models of beta diversity calculated with non-rarefied relative abundances versus each of the 100 independently rarefied abundances. Mean values of intercepts ($\overline{b}$) and slope ($\overline{m}$) and corresponding standard errors in parentheses are averages across the 100 regression models. The red line with intercept = 0 and slope = 1 represents a theoretical perfect agreement between beta diversity calculated with non-rarefied relative abundances and rarefied abundances.

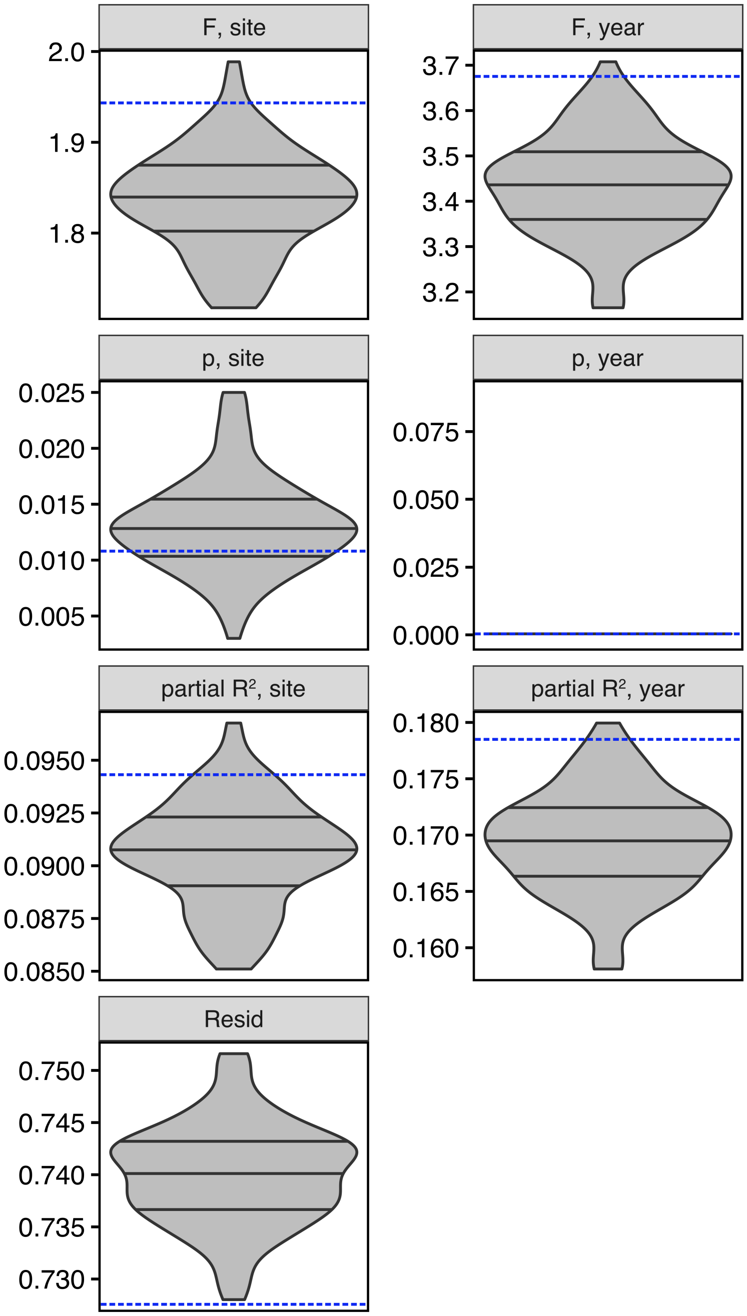

Figure S2: Violin plots showing distributions of partial R^2, F statistics, p values, and residuals of 100 PERMANOVA tests for differences in taxonomic microbial community composition between site and year based on Bray-Curtis dissimilarity calculated with rarefied ASV abundances from 100 independent iterations of random subsampling without replacement to an equal number of 214 reads per sample. Solid black lines represent quartiles (Q1, median, Q3). The blue dashed lines indicate values obtained from the analysis with non-rarefied relative ASV abundances presented in the main text. Only samples from Jungfraujoch and Sonnblick were considered since samples from Zugspitze were not available for both years.

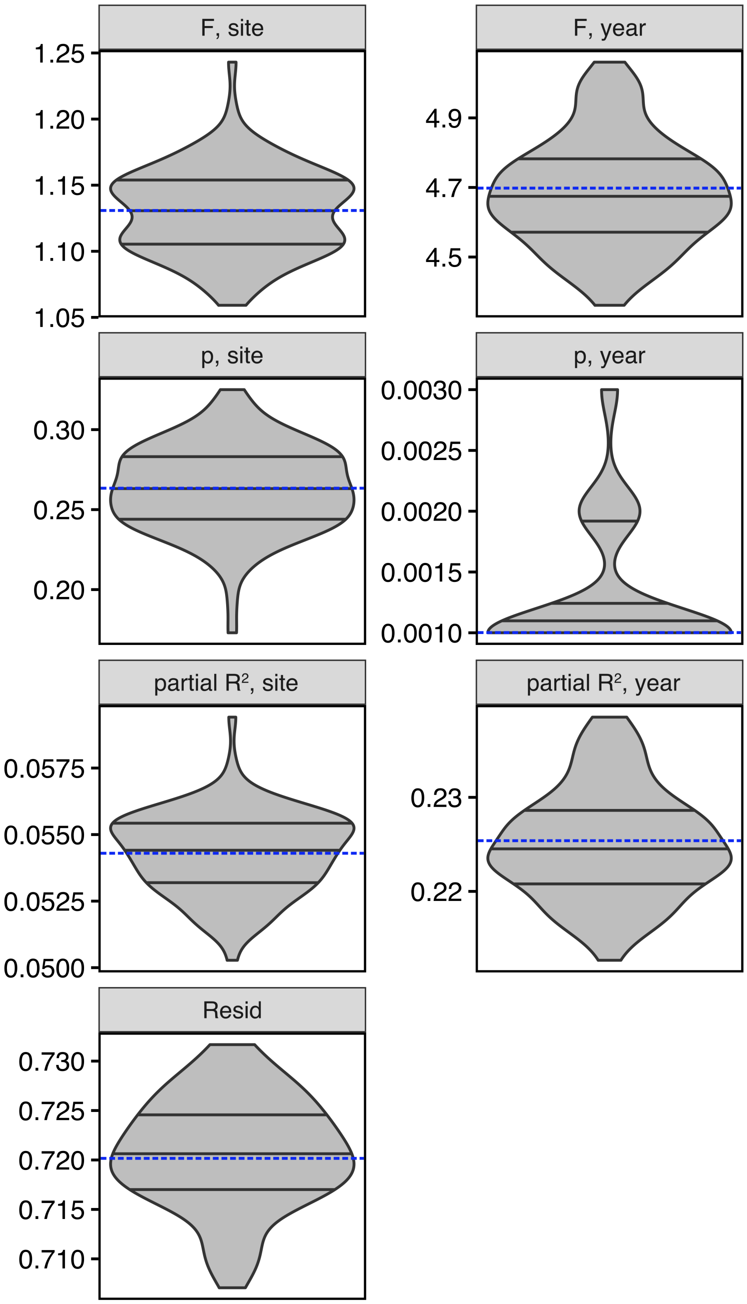

Figure S3: Violin plots showing distributions of partial R^2, F statistics, p values, and residuals of 100 PERMANOVA tests for differences in phylogenetic microbial community composition between site and year based on abundance-weighted β-MPD calculated with rarefied ASV abundances from 100 independent iterations of random subsampling without replacement to an equal number of 214 reads per sample. Solid black lines represent quartiles (Q1, median, Q3). The blue dashed lines indicate values obtained from the analysis with non-rarefied relative ASV abundances presented in the main text. Only samples from Jungfraujoch and Sonnblick were considered since samples from Zugspitze were not available for both years.

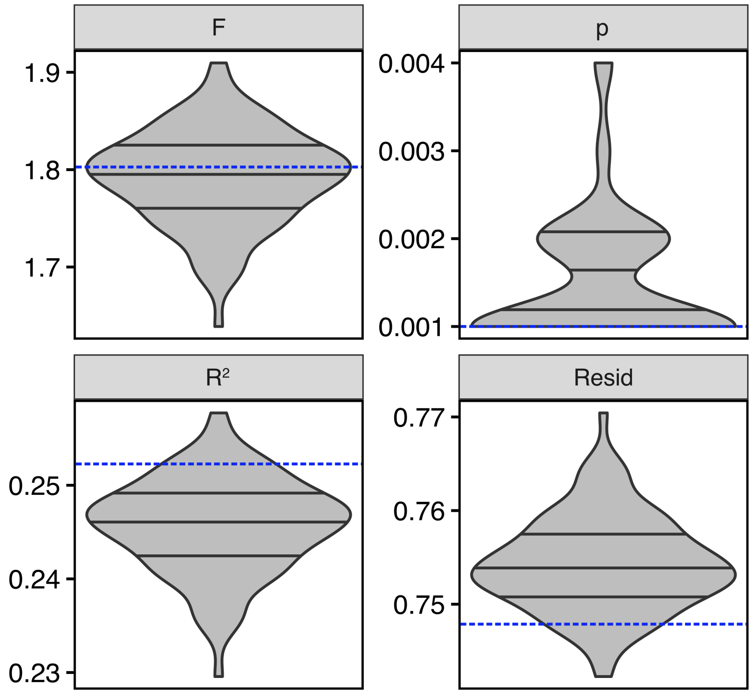

Figure S4: Violin plots showing distributions of R^2, F statistics, p values, and residuals of 100 PERMANOVA tests for differences in taxonomic microbial community composition between all three sites in 2016 based on Bray-Curtis dissimilarity calculated with rarefied ASV abundances from 100 independent iterations of random subsampling without replacement to an equal number of 214 reads per sample. Solid black lines represent quartiles (Q1, median, Q3). The blue dashed lines indicate values obtained from the analysis with non-rarefied relative ASV abundances presented in the main text.

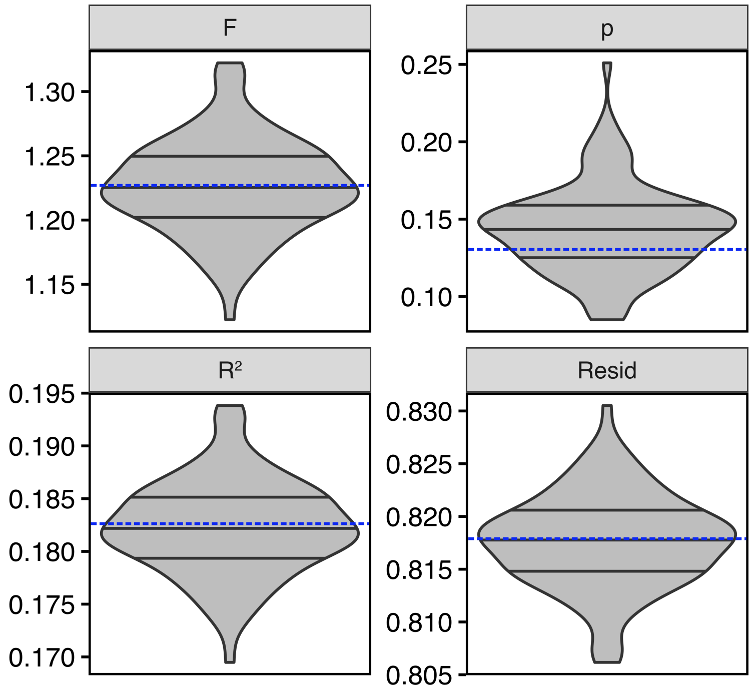

Figure S5: Violin plots showing distributions of R^2, F statistics, p values, and residuals of 100 PERMANOVA tests for differences in phylogenetic microbial community composition between all three sites in 2016 based on abundance-weighted β-MPD calculated with rarefied ASV abundances from 100 independent iterations of random subsampling without replacement to an equal number of 214 reads per sample. Solid black lines represent quartiles (Q1, median, Q3). The blue dashed lines indicate values obtained from the analysis with non-rarefied relative ASV abundances presented in the main text.

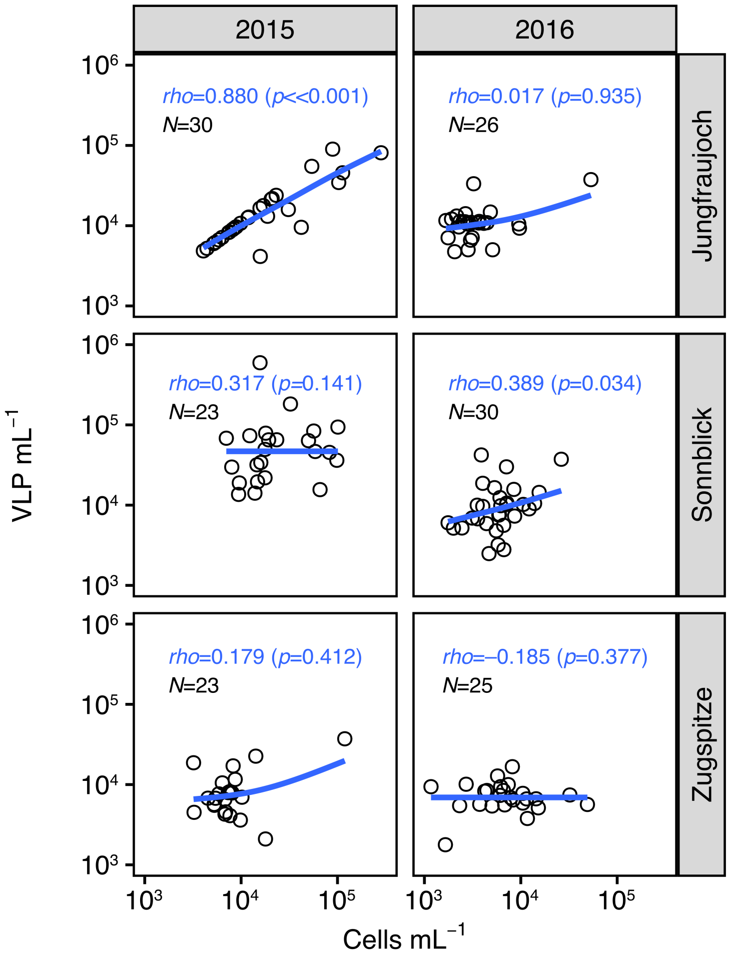

Figure S6: Correlations between numbers of prokaryotic cells and VLP per site and year. Fitted blue lines show trends from generalized additive model smoothing. Correlation coefficients were calculated as Spearman's rank correlations (*rho*).

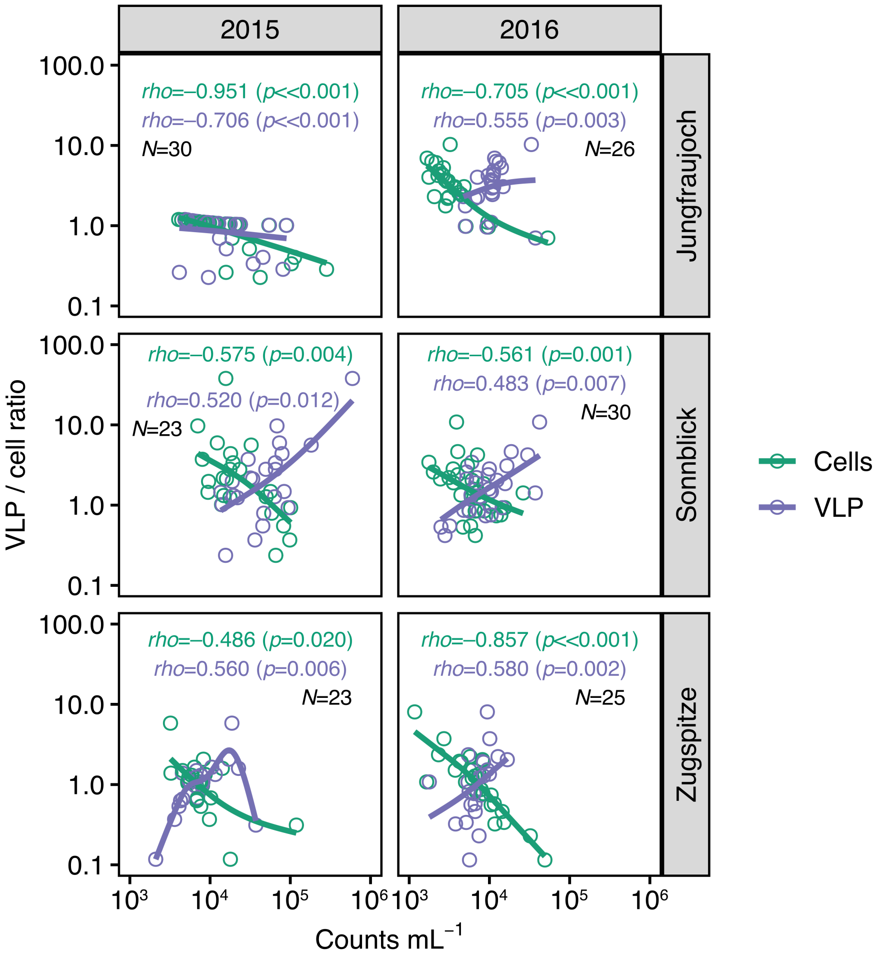

Figure S7: Correlations between VLP-to-prokaryotic cell ratios and numbers of prokaryotic cells and VLP, respectively, per site and year. Fitted lines show trends from generalized additive model smoothing. Correlation coefficients were calculated as Spearman's rank correlations (*rho*).

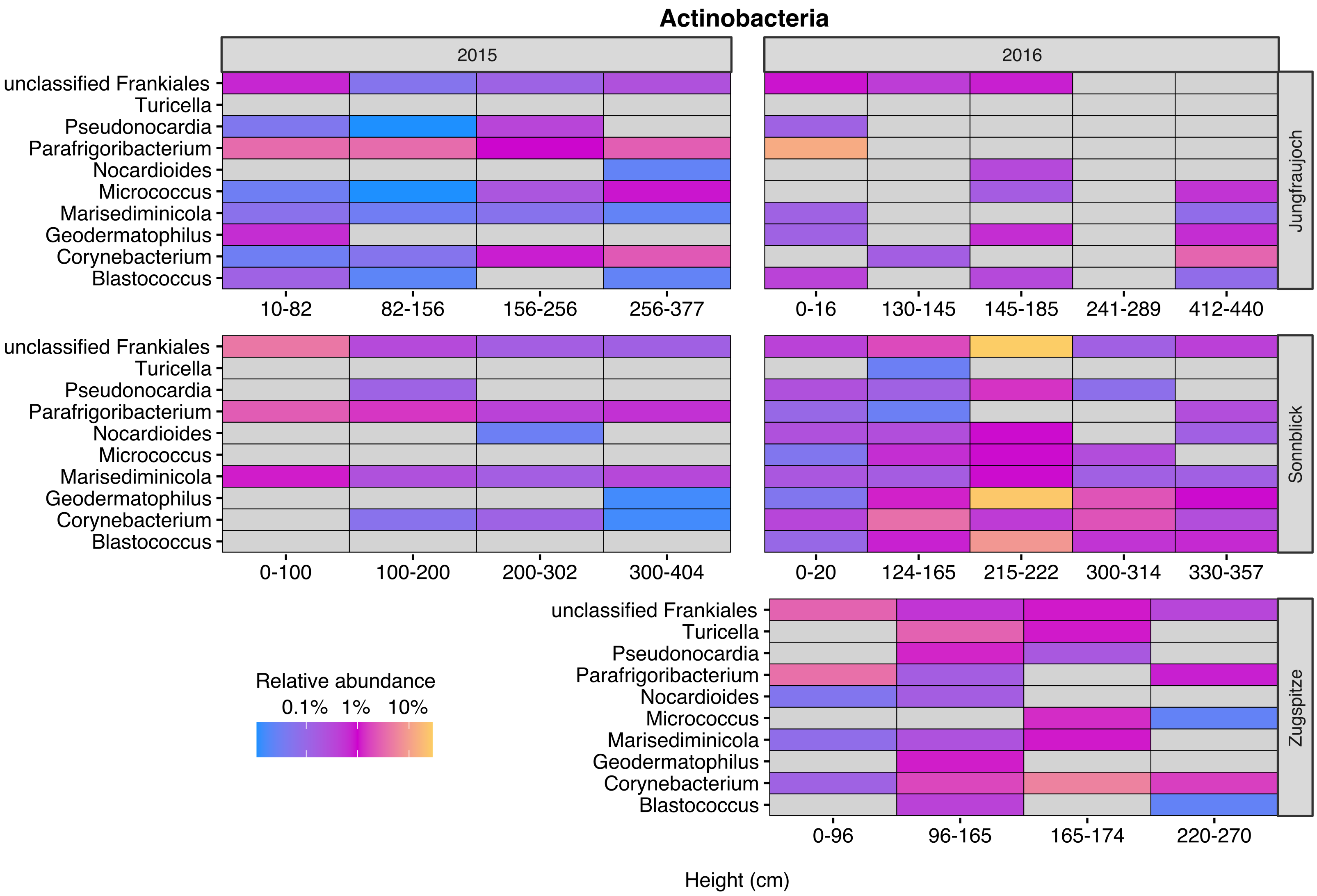

Figure S8: Heatmap showing abundances of the 10 most abundant genera within Actinobacteria across snowpack profiles per site and year. The range of pooled layers used for DNA extraction is indicated in cm above the bottom of the snowpack.

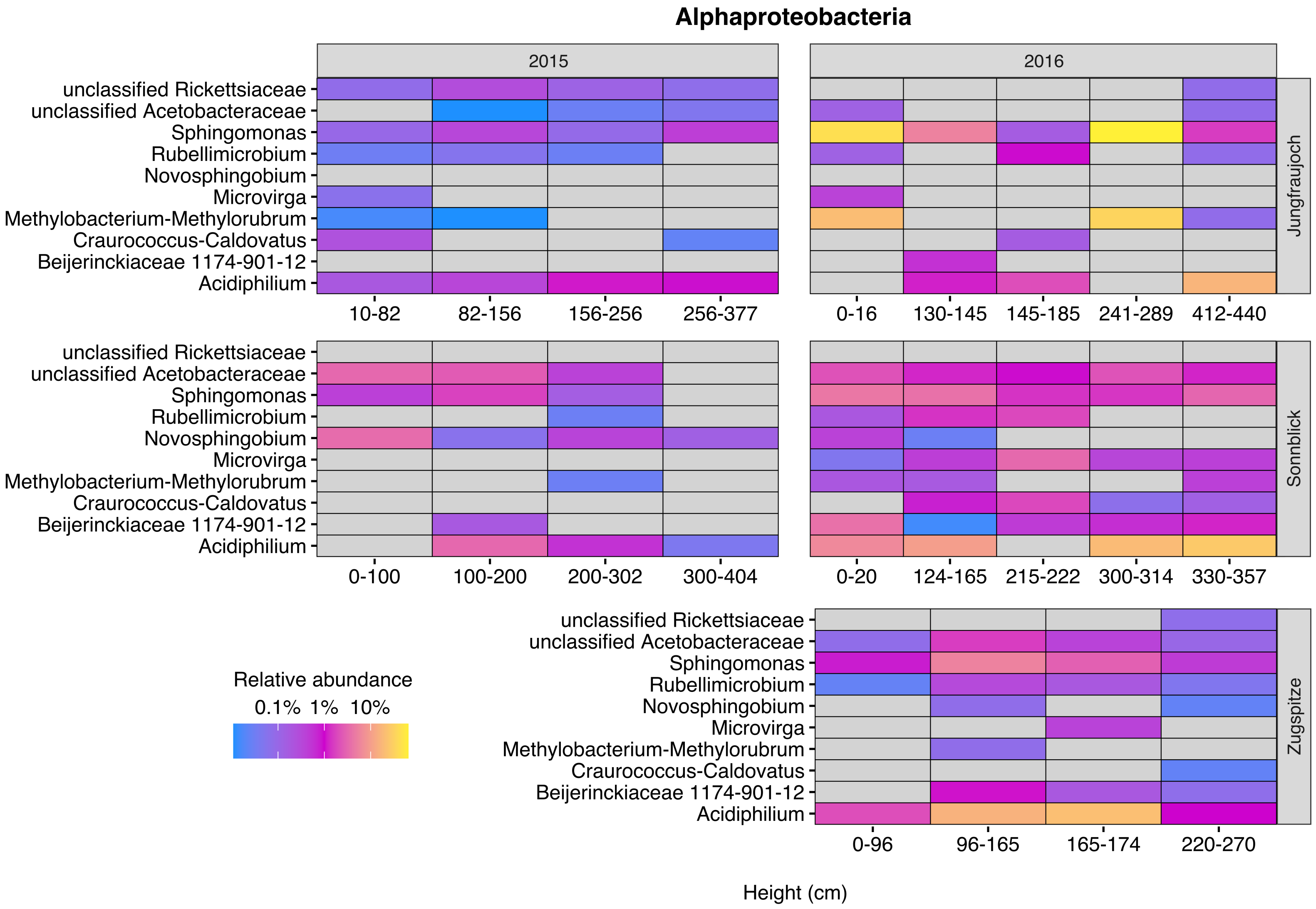

Figure S9: Heatmap showing abundances of the 10 most abundant genera within Alphaproteobacteria across snowpack profiles per site and year. The range of pooled layers used for DNA extraction is indicated in cm above the bottom of the snowpack.

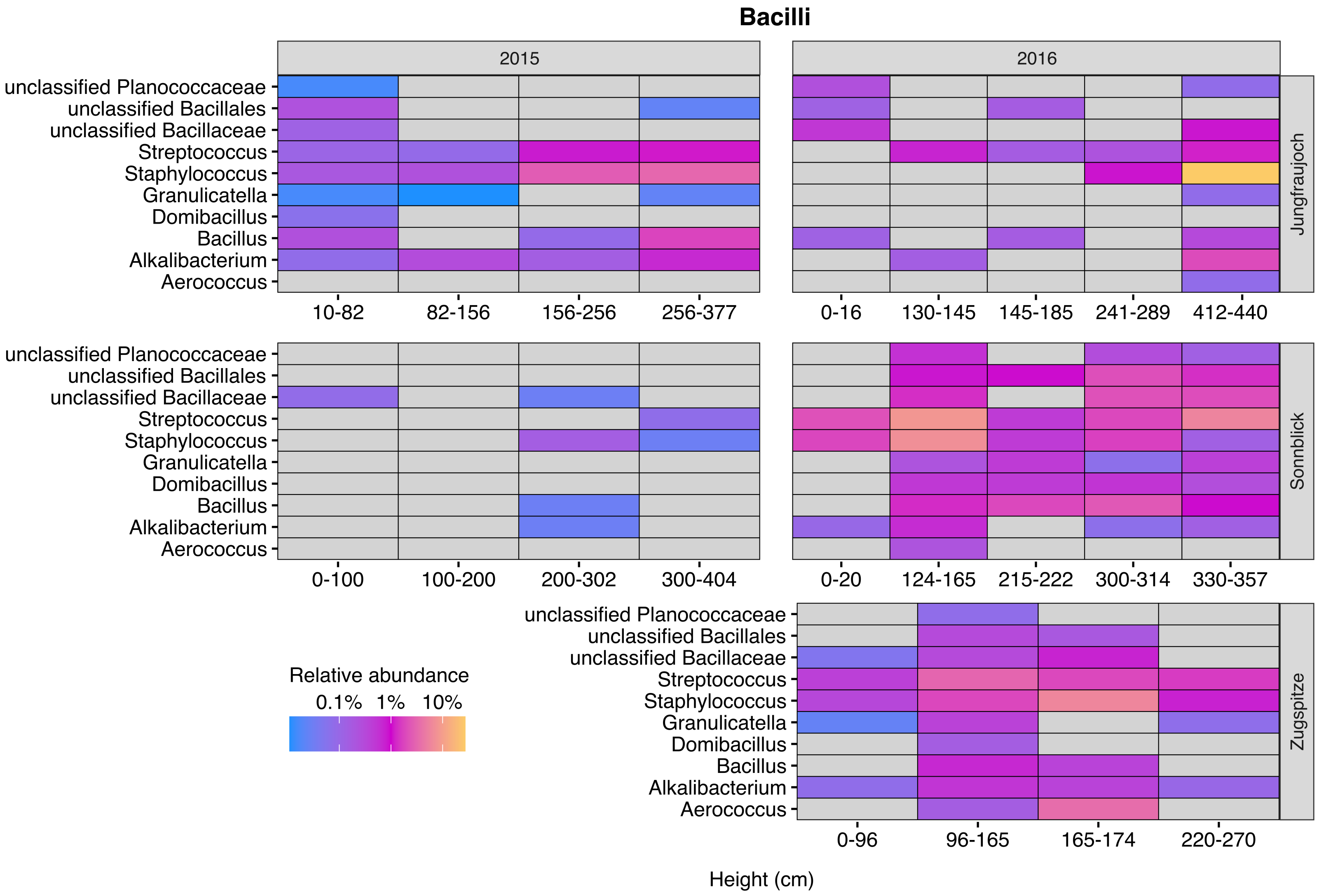

Figure S10: Heatmap showing abundances of the 10 most abundant genera within Bacilli across snowpack profiles per site and year. The range of pooled layers used for DNA extraction is indicated in cm above the bottom of the snowpack.

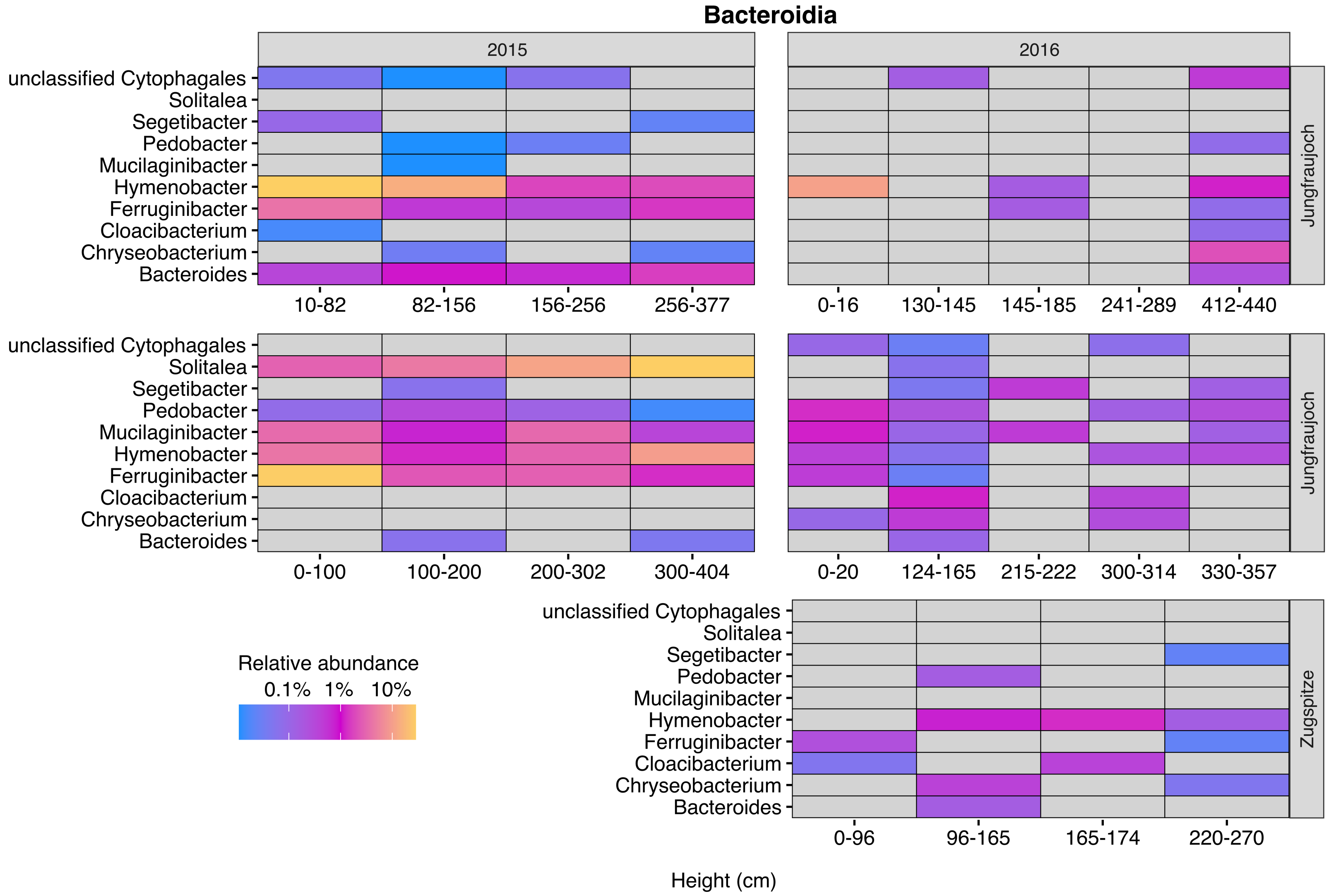

Figure S11: Heatmap showing abundances of the 10 most abundant genera within Bacteroidia across snowpack profiles per site and year. The range of pooled layers used for DNA extraction is indicated in cm above the bottom of the snowpack.

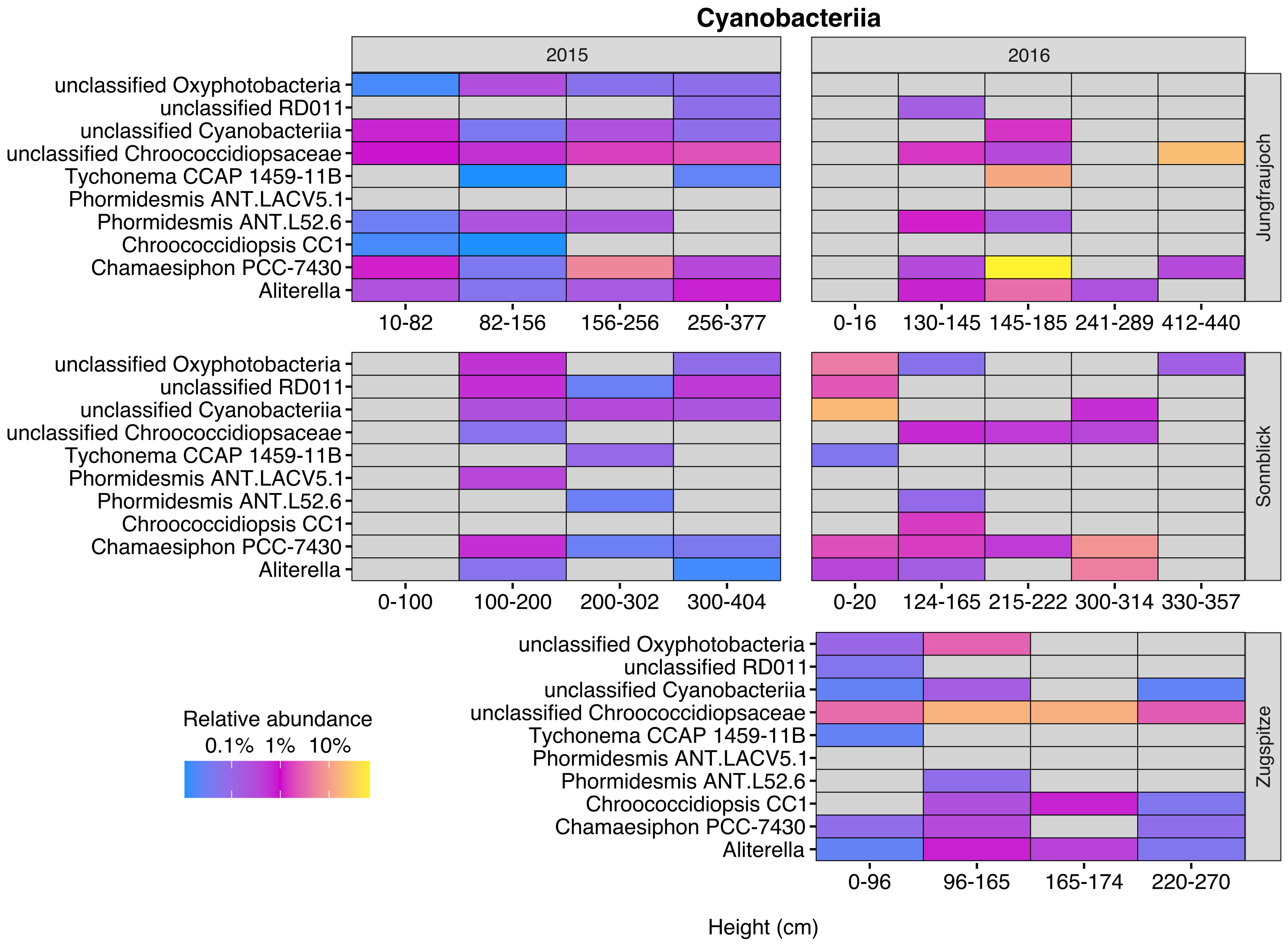

Figure S12: Heatmap showing abundances of the 10 most abundant genera within Cyanobacteriia across snowpack profiles per site and year. The range of pooled layers used for DNA extraction is indicated in cm above the bottom of the snowpack.

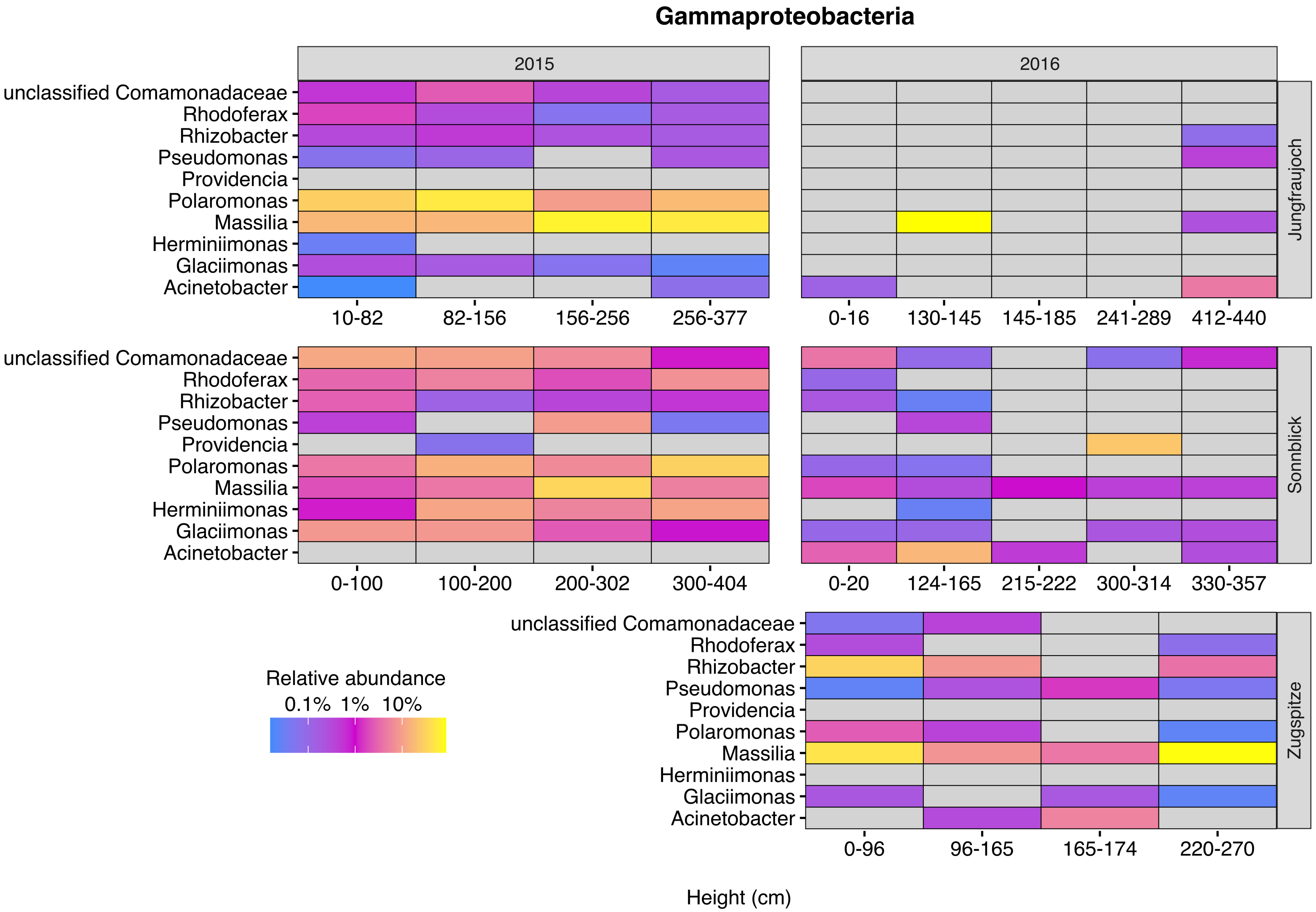

Figure S13: Heatmap showing abundances of the 10 most abundant genera within Gammaproteobacteria across snowpack profiles per site and year. The range of pooled layers used for DNA extraction is indicated in cm above the bottom of the snowpack.

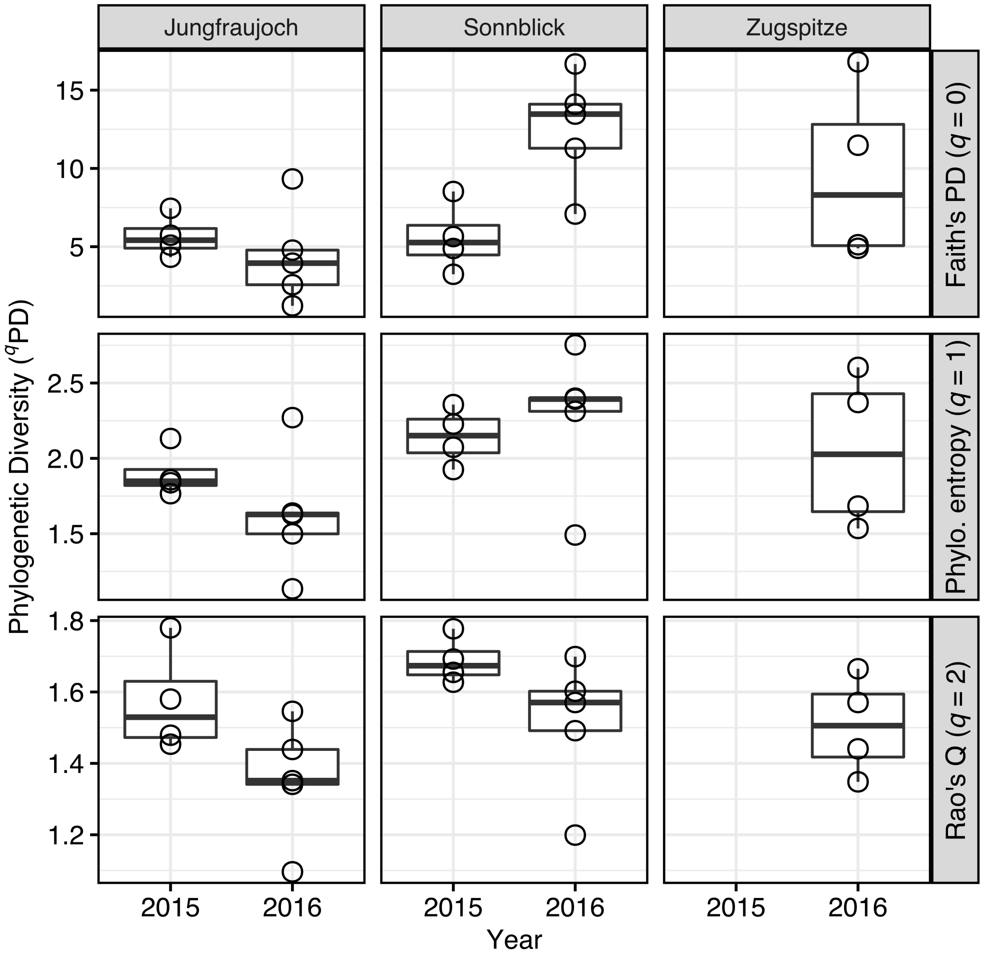

Figure S14: Estimates of phylogenetic microbial community alpha diversity inferred from Hill numbers per site and year.

Supplementary Tables

Table S1: Description of the snow layers according to Fierz et al. (2009), snow water equivalent (SWE), and snow density for Zugspitze, Germany, on May 13th 2015. Snow heights are given from 0 cm at the bottom (profile base) to maximum profile height at the snowpack surface. SWE and snow densities were measured directly in the field by determination of the snow mass of a defined volume of snow using a snow cylinder.

Snow height (cm)	Snow description	Snow density (kg m^{-3})	SWE (mm)
0-76	The rock surface builds the base of the snowpack at 0 cm. Snow grains are rounded, ~3 mm in diameter at the bottom, 2 mm at the top of the layer, slightly melted at the surfaces, snowpack medium hard, 0°C, wet at the snow base, moist at the top of the layer.	0-47 cm: 440<br>47-76 cm: 491	198<br>155
76-127	Snow grains rounded, ~3 mm in diameter, slightly melted at the surfaces, snowpack medium hard, 0°C, moist.	567	289
127-161	Snow grains rounded, ~3 mm in diameter, slightly melted at the surfaces, snowpack medium hard, 0°C, moist, ice layer between 127.0-127.5 cm.	529	180
161-175	Snow grains rounded, ~3.5 mm in diameter, slightly melted at the surfaces, snowpack very hard, 0.1°C, wet, two compact ice layers between 161-162 cm and again between 168-169 cm, single ice layer at 172-173 cm and ice lenses at 174 cm.	557	162
175-190	Snow grains rounded, ~3 mm in diameter, slightly melted at the surfaces, snowpack medium hard to hard, 0.1°C, moist, from 175 cm to top of the layer several ice lenses.		
190-206	Snow grains rounded, ~2 mm in diameter, slightly melted at the surfaces, snowpack medium hard, 0.1°C, moist, ellipsoid ice lenses between 201-206 cm.	546	158
206-219	Snow grains rounded, ~2 mm in diameter, slightly melted at the surfaces, snowpack weak, 0.1°C, moist, three thin ice layers of 1-2 mm thickness at 219 cm.		
219-233	Snow grains rounded, ~4 mm in diameter, slightly melted at the surfaces, snowpack very weak, 0.3°C, wet.	551	77

Table S2: Description of the snow layers according to Fierz et al. (2009), snow water equivalent (SWE), and snow density for Sonnblick, Austria, on July 2nd 2015. Snow heights are given from 0 cm at the bottom (profile base) to maximum profile height at the snowpack surface. SWE and snow densities were measured directly in the field by determination of the snow mass of a defined volume of snow using a snow cylinder (table continues on the next page).

Snow height (cm)	Snow description	Snow density (kg m^{-3})	SWE (mm)
0-25	Altered snow, grains are rounded, ~1-1.5 mm in diameter, slightly melted at the surfaces, partly clustered, snowpack hard to very hard, -0.25°C, slightly moist, lense-shaped ice layers of max. 2 mm thickness.	682	171
25-26	Compact ice layer, sometimes interrupted.		
26-50	Altered snow, grains are rounded, ~1 mm in diameter, slightly melted at the surfaces, partly clustered, snowpack very hard, -0.2°C, slightly moist, vertical ice lenses, layer comparable to lowermost layer.		
50-52	Compact ice layer.		
52-63	Altered snow, grains are rounded, ~1 mm in diameter, slightly melted at the surfaces, snowpack very hard, -0.2°C, slightly moist, very compact layer, very thin ice layers.		
63-75	Snow grains slightly rounded, 0.5-1 mm in diameter, coarsening to the top, slightly melted at the surfaces, snowpack very hard, 0°C, slightly moist, 6-8 continuous but braided 0.5-1.5 cm thick ice layers in small distances to each other.	701	84
75-97	Altered snow, grains rounded, 2-2.5 mm in diameter, slightly melted at the surfaces, partly clustered, snowpack hard to very hard, -0.1°C, slightly moist.		
97-100	Ice layer, slightly melted, compact, grains of 1.5-2 mm visible, slightly moist, -0.3°C, thin layers in short distances to each other.		

Table S2: Continued.

Snow height (cm)	Snow description	Snow density (kg m^{-3})	SWE (mm)
100-158	Snow grains rounded, <1 mm in diameter, slightly melted at the surfaces, snowpack medium hard to hard, -0.1°C, slightly moist, single very thin ice layers.	669	388
158-165	Compact ice layer with varying thickness of 1-6 cm.		
165-177	Snow grains rounded, 0.5-1 mm in diameter, slightly melted at the surfaces, snowpack medium hard to hard, 0°C, slightly moist.	547	66
177-178	Compact ice layer		
178-194	Snow grains rounded, 0.5 mm in diameter, slightly melted at the surfaces, snowpack hard, 0°C, slightly moist.	550	88
194-195	Compact ice layer.		
195-217	Snow grains rounded, 0.3-0.5 mm in diameter, slightly melted at the surfaces, snowpack medium hard, -0.1°C, slightly moist.	588	129
217-222	2-3 ice layers, very close together.		
222-337	Snow grains rounded, 0.5-1 mm in diameter, slightly melted at the surfaces, snowpack hard, -0.3°C, slightly moist, 2 cm ice lense at 280 cm, several coarse-grained, not continuous 1 mm thin layers, remaining layer very homogenous.	554	83
337-340	Compact ice layer.		
340-374	Snow grains rounded, 0.5 mm in diameter, slightly melted at the surfaces, snowpack medium hard, -0.1°C, slightly moist to moist, several thin ice layers.	904	307
374-391	Snow grains rounded, 0.5 mm in diameter, slightly melted at the surfaces, snowpack weak, 0°C, moist.	507	86
391-396	Dark, slightly brown layer, snow grains rounded, slightly melted at the surfaces, partly clustered, 2-3 mm in diameter, snowpack very weak, 0°C, moist, old surface assumed.	549	27
396-404	Snow grains rounded, ~1 mm in diameter, slightly melted at the surfaces, snowpack very weak, 0°C, moist.	566	45

Table S3: Description of the snow layers according to Fierz et al. (2009), snow water equivalent (SWE), and snow density for Jungfraujoch, Switzerland, on June 25th 2015. Snow heights are given from 0 cm at the bottom (profile base) to maximum profile height at the snowpack surface. SWE and snow densities were measured directly in the field by determination of the snow mass of a defined volume of snow using a snow cylinder (table continues on the next page).

Snow height (cm)	Snow description	Snow density (kg m^{-3})	SWE (mm)
0-10	A massive ice layer with an irregularly undulating surface builds the base of the snowpack at 0-10 cm.		
10-30	Snow grains are rounded, ~3 mm in diameter, slightly melted at the surfaces, snowpack hard to very hard, 0°C, moist.	553	122
30-32	Compact ice layer, irregular thickness of 1-2 cm, inclination in direction of the glacier.		
32-68	Crystal structure of the snow grains still partly recognizable, ~2 mm in diameter, slightly melted at the surfaces, snowpack hard to very hard, 0°C, moist. Layer can be divided in two very hard parts at the top and at the bottom and hard snowpack between 37-65 cm.	588	212
68-69	Compact ice layer.		
69-82	Snow grains slightly rounded, 3 mm in diameter, slightly melted at the surfaces, snowpack very hard, 0°C, moist.	566	74
82-148	Snow crystal structure still partly recognizable, 2 mm in diameter, slightly melted at the surfaces, snowpack hard, 0°C, slightly moist, at 100 cm, 120 cm and 139 cm non-continuous ice layers of max. 2 mm thickness.		
148-149	Ice layer.	578	428
149-155	Snow crystal structure still partly recognizable, 2 mm in diameter, slightly melted at the surfaces, snowpack hard, 0°C, slightly moist to moist, 1 mm ice layer at 153 cm.		
155-156	Ice layer.		

Table S3: Continued.

Snow height (cm)	Snow description	Snow density (kg m^{-3})	SWE (mm)
156-256	Snow grains slightly rounded, 1-2 mm in diameter, slightly melted at the surfaces, snowpack hard, 0°C, slightly moist. 36 ice layers, partly continuous, partly as lenses, max. 2 mm thickness, continuous ice layer of 1 cm thickness at 252-253 cm.	532	532
256-261	Yellowish-brown layer, Saharan dust? Snow grains are rounded, 3-4 mm in diameter, slightly melted at the surfaces, snowpack weak, 0°C, wet.	454	23
261-314	Snow grains rounded, 3 mm in diameter, slight tendency to coarsening-up towards the top, slightly melted at the surfaces, snowpack very weak, 0.1°C, wet and snow grains covered by water film.	551	375
314-329	Snow grains rounded, 3 mm in diameter, slightly melted at the surfaces, snowpack weak, 0.1°C, wet, harder icy parts in the upper part of the layer.		
329-347	Snow grains rounded, 2 mm in diameter, less coarse and harder than in the layers below, slightly melted at the surfaces, snowpack medium hard, 0.1°C, moist.	401	136
347-363	Snow crystal structure still partly recognizable, 1-2 mm in diameter, slightly melted at the surfaces, snowpack medium hard, moist, features of an altered fresh snow layer, very thin ice layers at 358 cm.		
363-372	Snow grains rounded, 2-3 mm in diameter, slightly melted at the surfaces, snowpack hard and moist, covered by harsh, 3-4 ice layers of 1 mm thickness between 272-273 cm.	353	49

Table S4: Description of the snow layers according to Fierz et al. (2009), snow water equivalent (SWE), and snow density for Zugspitze, Germany, on May 3rd 2016. Snow heights are given from 0 cm at the bottom (profile base) to maximum profile height at the snowpack surface. SWE and snow densities were measured directly in the field by determination of the snow mass of a defined volume of snow using a snow cylinder.

<table>
<tr><th>Snow height (cm)</th><th>Snow description</th><th>Snow density (kg m^{-3})</th><th>SWE (mm)</th></tr>
<tr><td>0-103</td><td>The rock surface builds the base of the snowpack at 0 cm. Altered snow, grains are rounded, 1-2 mm in diameter, snowpack very hard, 0°C, dry.</td><td>498</td><td>513</td></tr>
<tr><td>103-104</td><td>Ice layer.</td><td></td><td></td></tr>
<tr><td>104-115</td><td>Altered snow, grains are rounded, 1-2 mm in diameter, snowpack very hard, 0°C, dry.</td><td>556</td><td>61</td></tr>
<tr><td>115-174</td><td>Altered snow, grains are rounded, ~2 mm in diameter, snowpack hard, 0.1°C, several thin ice layers, 2 cm ice layer at the snow base.</td><td>507</td><td>299</td></tr>
<tr><td>174-184</td><td>Floating snow, brownish, Saharan dust event proven on April 6th, 2016, grains are rounded, 2-3 mm in diameter, snowpack hard, 0°C, slightly moist.</td><td>589</td><td>59</td></tr>
<tr><td>184-196</td><td>Altered snow, grains slightly rounded, 1 mm in diameter, snowpack medium hard, 0°C, dry.</td><td rowspan="3">435</td><td rowspan="3">135</td></tr>
<tr><td>196-205</td><td>Altered snow, grains are rounded, 1 mm in diameter, snowpack hard, 0°C, dry, several ice layers with max. 5 mm thickness.</td></tr>
<tr><td>205-215</td><td>Altered snow, grains are rounded, relatively coarse, 2-3 mm in diameter, somewhen before slightly melted, snowpack hard, 0°C, slightly moist.</td></tr>
<tr><td>215-230</td><td>Altered snow, grains are slightly rounded, structure of broken crystals is still recognizable, 1-2 mm in diameter, snowpack weak, 0°C, dry.</td><td>389</td><td>58</td></tr>
<tr><td>230-238</td><td>Fresh snow, harsh layer at the surface, crystal structure is still recognizable, grains are only very slightly rounded, 2 mm in diameter, snowpack weak and dry, -0.2°C, ice layer of 1-2 cm and two other ones with 5 mm thickness.</td><td>303</td><td>24</td></tr>
<tr><td>238-270</td><td>Fresh snow of the last 3 d, snow crystals still recognizable, rimed, 1 mm in diameter, snowpack very weak and dry, -0.9°C.</td><td>214</td><td>68</td></tr>
</table>

Table S5: Description of the snow layers according to Fierz et al. (2009), snow water equivalent (SWE), and snow density for Sonnblick, Austria, on June 8th 2016. Snow heights are given from 0 cm at the bottom (profile base) to maximum profile height at the snowpack surface. SWE and snow densities were measured directly in the field by determination of the snow mass of a defined volume of snow using a snow cylinder (table continues on the next page).

**Snow height (cm)**	**Snow description**	**Snow density ($kg\ m^{-3}$)**	**SWE (mm)**
0-20	The rock surface builds the base of the snowpack at 0 cm. Altered snow, facetted crystals, grains are rounded and rimed, coarse, ~3 mm in diameter, snowpack medium hard, -1.5°C, slightly moist.	379	190
20-22	Compact ice layer, -1.4°C, dry.		
22-50	Altered snow, facetted crystals, 2 mm in diameter, snowpack weak and dry, -1.2°C.		
50-123	Altered snow, fine crystals, broken or slightly rounded, 1 mm in diameter, snowpack very hard, -0.5°C, dry.	50-104 cm: 495, 104-123 cm: 497	50-104 cm: 267, 104-123 cm: 94
123-124	Compact ice layer, -0.1°C, dry.		
124-165	Altered snow, crystals are broken or slightly rounded, 1 mm, snowpack very hard, 0°C, dry, at 146 cm, 156 cm and 164 cm ice layers up to 1 cm thickness.	124-147 cm: 554, 147-171 cm: 507	124-147 cm: 127, 147-171 cm: 122
165-172	Several non-continuous ice layers up to 1 cm thick, altered snow inbetween, crystals broken or slightly rounded, 1 mm in diameter, snowpack very hard, 0°C, dry.		
172-215	Altered snow, grains are rounded, slightly greyish, slightly melted at the surfaces, 1 mm in diameter, snowpack medium hard to hard, 0°C, dry, ice layer at 200-201 cm.	171-201 cm: 499, 201-215 cm: 488	171-201 cm: 150, 201-215 cm: 68

Table S5: Continued.

Snow height (cm)	Snow description	Snow density (kg m^{-3})	SWE (mm)
215-227	Altered snow, brown, grains are rounded, 1 mm in diameter, snowpack medium hard, 0°C, slightly moist, Saharan dust event proven on April 4th, 2016.	328	39
227-240	Altered snow, inclined position of single lamina, grains are rounded, 2 mm in diameter, snowpack weak to medium hard, 0°C, dry to slightly moist.	458	60
240-250	Altered snow, grains are rounded, 2 mm in diameter, snowpack weak, 0°C, slightly moist, several ice layers of max. 5 mm thickness.		
250-267	Altered snow, grains are rounded, 2 mm in diameter, snowpack medium hard, 0°C, dry.	250-271 cm: 475	250-271 cm: 100
267-300	Altered snow, crystals are broken or slightly rounded, ~2 mm in diameter, snowpack weak and dry, 0.1°C, thin greyish layers.	271-300 cm: 413	271-300 cm: 120
300-314	Altered snow, grains are rounded, ~2 mm in diameter, snowpack hard, 0.1°C, slightly moist, thin greyish layers.	432	60
314-328	Altered snow, grains are rounded, ~1 mm in diameter, snowpack hard, 0.1°C, slightly moist.	472	66
328-336	Fresh snow, grains are rounded, slightly brownish-grey, 2 mm in diameter, snowpack very weak, 0°C, slightly moist.	365	29
336-357	Fresh snow, crystal shape still recognizable, 1 mm in diameter, snowpack very weak, 0.1°C, dry.	251	53

Table S6: Description of the snow layers according to Fierz et al. (2009), snow water equivalent (SWE), and snow density for Jungfraujoch, Switzerland, on June 1st 2016. Snow heights are given from 0 cm at the bottom (profile base) to maximum profile height at the snowpack surface. SWE and snow densities were measured directly in the field by determination of the snow mass of a defined volume of snow using a snow cylinder (table continues on the next page).

Snow height (cm)	Snow description	Snow density (kg m^{-3})	SWE (mm)
0-16	Altered snow, single crystals of ~1 mm diameter, snowpack very hard, -6.1°C, dry.	410	66
16-20	Altered snow, slightly brown (Saharan dust?), singly crystals of ~1 mm diameter, snowpack very hard, -6.0°C, dry.		
20-117	Altered snow, singly crystals of ~1 mm diameter, snowpack very hard, -4.5°C, dry.	446	433
117-130	Altered snow, crystals slightly rounded, ~1 mm in diameter, snowpack very hard, -3.2°C, dry. 3-4 ice layers inbetween.	554	72
130-145	Altered snow, brown (Saharan dust clearly recognizable), fuzzy boundary to the top and the underlying snow, singly crystals of ~1 mm diameter, snowpack very hard, -4.2°C, at the top and at the bottom 3-4 cm layer with several ice layers.	404	61
145-185	Altered snow, single crystals of ~1 mm diameter, snowpack very hard, -4.0°C, dry.	504	202
185-211	Altered snow, non-continuous dust lense, maybe redistributed, snow grains are slightly rounded, 1 mm in diameter, snowpack very hard, -3.1°C, dry.	481	125
211-237	Altered snow, ice blocks visible, snow grains slightly rounded, 2 mm in diameter, snowpack hard, -2.5°C, dry.	421	109
237-241	Altered snow, varying thickness of the undulating layer, snow grains are rounded, very coarse, 2 mm in diameter, formerly melted, snowpack slightly greyish-brown (Saharan dust?), hard, -3.0°C, dry.		

Table S6: Continued.

Snow height (cm)	Snow description	Snow density (kg m^{-3})	SWE (mm)
241-289	Altered snow, grains are slightly rounded, ~1 mm in diameter, snowpack very hard, dry, -2.3°C, non-continuous ice layers of <0.5 mm thickness.	418	201
289-300	Altered snow, grains are slightly rounded to rounded, ~2 mm in diameter, snowpack very hard, dry, -1.8°C, 2 cm thick ice layer at the snow base.		
300-390	Altered snow, relative homogeneous, grains are rounded, 1-2 mm in diameter, snowpack medium hard, -0.1°C at the top, 0.7°C in the middle and -2.0°C at the snow base, dry, several layers of 1-2 cm with coarser grains (2 mm) and weaker snowpack.	341	307
390-412	Snow grains are slightly rounded, 2 mm in diameter, snowpack weak, 0.1°C, dry.	387	85
412-433	Snow grains are rounded, 3 mm in diameter, melted at the surfaces, snowpack very weak, 0.2°C, slightly moist.	424	30
433-440	Harsh, snow grains are rounded, 3 mm in diameter, snowpack medium hard, 0.2°C, dry.		

Table S7: Isotope ratios in precipitation and precipitation amounts (Precip.) per location (table continues on the next page).

	Jungfraujoch[a]			Sonnblick[c]			Zugspitze[d]		
	δ^2H (‰)	$\delta^{18}O$ (‰)	Precip. (mm)[b]	δ^2H (‰)	$\delta^{18}O$ (‰)	Precip. (mm)	δ^2H (‰)	$\delta^{18}O$ (‰)	Precip. (mm)
Sep 2014	-79.4	-11.6	79	-77.6	-11.7	98			
Oct 2014	-77.9	-11.8	132	-86.7	-12.5	76	-69.1	-11.0	159
Nov 2014	-149.9	-20.0	169	-128.8	-17.5	163	-125.0	-16.9	64
Dec 2014	-144.9	-19.7	172	-108.3	-15.1	71	-168.0	-22.0	206
Jan 2015	-144.2	-19.4	281	-86.8	-12.3	149	-121.7	-16.5	249
Feb 2015	-159.8	-21.5	147	-106.6	-14.9	76	-121.1	-16.9	92
Mar 2015	-129.9	-17.6	280	-97.8	-13.5	144	-87.5	-12.3	238
Apr 2015	-128.9	-17.5	213	-74.8	-11.2	188	-91.5	-12.6	198
May 2015	-104.4	-14.1	198	-68.1	-10.1	179	-118.4	-15.9	41
Jun 2015	-79.8	-11.5	131	-58.2	-8.98	191			

Table S7: Continued

	Jungfraujoch[a]			Sonnblick[c]			Zugspitze[d]		
	δ^2H (‰)	$\delta^{18}O$ (‰)	Precip. (mm)[b]	δ^2H (‰)	$\delta^{18}O$ (‰)	Precip. (mm)	δ^2H (‰)	$\delta^{18}O$ (‰)	Precip. (mm)
Sep 2015	-74.4	-11.4	115	-72.4	-11.01	162			
Oct 2015	-117.2	-16.1	92	-125	-16.99	119			
Nov 2015	-166.3	-22.8	148	-102.6	-14.2	61	-155.1	-20.4	182
Dec 2015	-144.4	-20.0	25	-82.2	-11.29	11	-96.2	-13.6	68
Jan 2016	-140.6	-19.6	219	-117.5	-16.29	138	-127.2	-17.6	357
Feb 2016	-128.7	-17.8	232	-93.3	-12.92	193	-121.0	-16.2	330
Mar 2016	-156.4	-20.9	127	-142.1	-18.65	116	-116.7	-15.1	91
Apr 2016	-116.4	-16.3	211	-76.1	-11.35	161	-92.8	-12.5	148
May 2016	-111.9	-15.6	258	-82.5	-11.9	178	-67.6	-9.6	383
Jun 2016	-103.2	-14.3	146						

[a] Data from M. Leuenberger (personal communication, February 9th 2016), High Altitude Research Station Jungfraujoch.
[b] Data from M. Leuenberger (personal communication, February 9th 2016), closest weather station at Grimsel.
[c] Data from Feuerkogel being closest to Sonnblick (Environment Agency Austria, 2016).
[d] Data corrected from Hürkamp et al. (2019).

References

Environment Agency Austria (2016). Österreichisches Messnetz für Isotope im Niederschlag und in Oberflächengewässern (ANIP). Available at: https://www.umweltbundesamt.at/wasser/informationen/isotope/isotopenmessnetz-anip [Accessed May 12, 2016].

Fierz, C., Armstrong, R. L., Durand, Y., Etchevers, P., Greene, E., McClung, D. M., et al. (2009). The International Classification for Seasonal Snow on the Ground. *Technical Documents in Hydrology N°83, IACS Contribution N°1, UNESCO-IHP, Paris*.

Hürkamp, K., Zentner, N., Reckerth, A., Weishaupt, S., Wetzel, K.-F., Tschiersch, J., et al. (2019). Spatial and temporal variability of snow isotopic composition on Mt. Zugspitze, Bavarian alps, Germany. *J. Hydrol. Hydromech.* 67, 49–58. doi:10.2478/johh-2018-0019.
